# Dinaciclib synergizes with BH3 mimetics targeting BCL‐2 and BCL‐X_L_ in multiple myeloma cell lines partially dependent on MCL‐1 and in plasma cells from patients

**DOI:** 10.1002/1878-0261.13522

**Published:** 2023-09-28

**Authors:** Manuel Beltrán‐Visiedo, Nelia Jiménez‐Alduán, Rosana Díez, Marta Cuenca, Andrea Benedi, Alfonso Serrano‐Del Valle, Gemma Azaceta, Luis Palomera, Victor Peperzak, Alberto Anel, Javier Naval, Isabel Marzo

**Affiliations:** ^1^ Apoptosis, Immunity & Cancer Group, IIS Aragón University of Zaragoza Spain; ^2^ Hematology Service Hospital Universitario Miguel Servet Zaragoza Spain; ^3^ Center for Translational Immunology, University Medical Center Utrecht Utrecht University The Netherlands; ^4^ Hematology Service Hospital Clínico Universitario Lozano Blesa Zaragoza Spain; ^5^ HCU‐Lozano Blesa‐Hematology Research Group, IIS Aragón Instituto Aragonés de Ciencias de la Salud Zaragoza Spain

**Keywords:** BCL‐2 proteins, BH3 mimetics, CDK inhibitors, cell death, multiple myeloma

## Abstract

A better understanding of multiple myeloma (MM) biology has led to the development of novel therapies. However, MM is still an incurable disease and new pharmacological strategies are needed. Dinaciclib, a multiple cyclin‐dependent kinase (CDK) inhibitor, which inhibits CDK1, 2, 5 and 9, displays significant antimyeloma activity as found in phase II clinical trials. In this study, we have explored the mechanism of dinaciclib‐induced death and evaluated its enhancement by different BH3 mimetics in MM cell lines as well as in plasma cells from MM patients. Our results indicate a synergistic effect of dinaciclib‐based combinations with B‐cell lymphoma 2 or B‐cell lymphoma extra‐large inhibitors, especially in MM cell lines with partial dependence on myeloid cell leukemia sequence 1 (MCL‐1). Simultaneous treatment with dinaciclib and BH3 mimetics ABT‐199 or A‐1155463 additionally showed a synergistic effect in plasma cells from MM patients, *ex vivo*. Altered MM cytogenetics did not affect dinaciclib response *ex vivo*, alone or in combined treatment, suggesting that these combinations could be a suitable therapeutic option for patients bearing cytogenetic alterations and poor prognosis. This work also opens the possibility to explore cyclin‐dependent kinase 9 inhibition as a targeted therapy in MM patients overexpressing or with high dependence on MCL‐1.

## Graphical abstract

Multiple myeloma (MM) remains an incurable disease. As a general rule, MM cells overexpress or are highly dependent on MCL‐1 to survive. Therefore, dinaciclib (a multiple CDK inhibitor) decreases MM cells viability due to CDK9 inhibition and subsequent MCL‐1 downregulation. Rational combinations with corresponding BH3 mimetics in cases of codependence on BCL‐2 or BCL‐X_L_ notably reduce cell resistance to death.

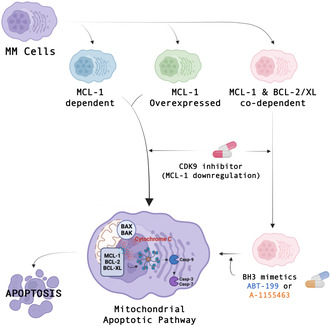

**Abbreviations**
+1q: gain/amplification of chromosome 1q
ABB: annexin V binding buffer
ALL: acute lymphocytic leukemia
AML: acute myeloid leukemia
AUC: area under the curve
BCL‐2: B‐cell lymphoma 2
BCL‐X_L_
: B‐cell lymphoma extra‐large
BM: bone marrow
BMMCs: BM mononuclear cells
CDK: cyclin‐dependent kinase
CLL: chronic lymphoid leukemia
CRISPR: clustered regularly interspaced short palindromic repeats
DBP: dynamic BH3 profiling
DKO: double KO
EXP: hypothetically expected specific apoptosis
FGFR3: fibroblast growth factor receptor 3
FISH: fluorescence *in situ* hybridization
HDAC: histone deacetylase
HSP90: heat shock protein 90
IC_50_
: half‐inhibitory concentration
IGH: immunoglobulin heavy chain
IL: interleukin
IMiDs: immunomodulatory drugs
KO: knock out
LD_50_
: lethal dose
MCL‐1: myeloid cell leukemia sequence 1
MGUS: monoclonal gammopathy of undetermined significance
MM: multiple myeloma
MOMP: mitochondrial outer membrane permeabilization
MTT: 3‐(4,5‐dimethylthiazol‐2‐yl)‐2,5‐diphenyl‐2H‐tetrazolium bromide
OBS: experimentally observed specific apoptosis
PCL: plasma cell leukemia
PS: phosphatidylserine
ROC: receiver operating characteristics
SMM: smoldering MM
T‐ALL: T‐cell acute lymphoblastic leukemia
TP53: tumor protein P53
zVAD: Z‐VAD‐FMK

## 1 Introduction

Multiple myeloma (MM) is currently an incurable hematological cancer whose origin is incompletely known. MM is characterized by the appearance of multiple malignant plasma cell foci in the bone marrow (BM) causing bone destruction, thrombocytopenia and neutropenia [1]. Virtually all patients with MM evolve from an asymptomatic premalignant disease stage known as monoclonal gammopathy of undetermined significance (MGUS). In some cases, smoldering MM (SMM), an asymptomatic but more advanced premalignant step, can be clinically recognized between MGUS and MM stages. Finally, and during the last stage of the disease, aberrant plasma cells can grow extramedullary and infiltrate other organs or tissues developing plasma cell leukemia (PCL) with the presence of ≥ 5% circulating plasma cells [2]. During the last two decades, the chemotherapy of MM has experienced significant improvements due to the discovery of more selective drugs like immunomodulatory drugs (IMiDs, such as lenalidomide, pomalidomide) and proteasome inhibitors (bortezomib, carfilzomib and ixazomib) that have better therapeutic efficacy and fewer side effects. However, although overall survival and patient response have considerably improved, most patients experience relapse after first‐line treatment and drug resistance is still a major concern that accounts for the fatality of the disease [3]. Therefore, novel drugs with an improved pharmacological profile are still needed.

Cyclin‐dependent kinases (CDKs) are serine/threonine kinases that regulate progression through cell cycle, by means of complexes with specific cell cycle regulatory proteins called cyclins [4]. Aside from being the main core of cell cycle regulatory machinery (e.g. CDK1, 2, 4 and 6), CDKs also regulate other cell processes such as mRNA splicing (CDK11 and 13) or protein transcription (CDK7–9, 12 and 13) [5, 6, 7, 8, 9]. Other CDKs (CDK10, 11 and 14–18) have unique functions that are frequently tissue specific [6]. Among others, dysregulation of cyclin D (and therefore that of CDK4 and 6) occurs in virtually all MM cells [4, 10]. For this reason, CDK inhibitors have been proposed as good candidates for MM therapy [10]. However, several clinical trials based on the first‐generation CDK inhibitors, such as flavopiridol and roscovitine, have been ended before their completion due to low antitumor activity and adverse effects [11, 12]. In this regard, second generation of CDK inhibitors (e.g. dinaciclib, AT7519 and fadraciclib) were developed to improve their pharmacological profile. Additionally, there was an additional third generation of CDK inhibitors (mostly CDK4/6 inhibitors). Intriguingly, some members of this last generation (e.g. palbociclib, ribociclib, abemaciclib and trilaciclib) were FDA approved for first‐ or second‐line treatments of patients with hormone receptor‐positive, advanced or metastatic breast cancer and lung cancer [13, 14, 15].

Dinaciclib is an ATP‐competitive inhibitor of CDK1, 2, 5 and 9 [16] with a half‐inhibitory concentration (IC_50_) in the low nanomolar range [4]. It has demonstrated, alone or in combination with proteasome inhibitors, anti‐MM activity in phase II clinical trials [4, 5]. Preclinically, dinaciclib prevented cell cycle progression, drastically reduced MCL‐1 protein expression and caused the regression of implanted solid tumors in a variety of xenograft models [17, 18]. Clinically, it has demonstrated anti‐MM activity in phase II clinical trial (NCT01096342) [4]. The most frequent toxicities detected across this study were leukopenia, thrombocytopenia, nausea, diarrhea and fatigue.

Most types of cancer, including MM, avoid cell death by overexpressing diverse antiapoptotic proteins of the BCL‐2 family [e.g. B‐cell lymphoma 2 (BCL‐2), myeloid cell leukemia sequence 1 (MCL‐1) and B‐cell lymphoma extra‐large (BCL‐X_L_)]. These proteins sequester proapoptotic members of the same family inhibiting mitochondrial outer membrane permeabilization (MOMP) [19], the key step in the intrinsic apoptotic pathway activated by most antitumoral drugs. Advances in the knowledge of the structure and mechanism of action of antiapoptotic and proapoptotic members of the BCL‐2 family has allowed the development of the so‐called BH3 mimetics, small molecules acting as BH3 agonists [19, 20]. BH3 mimetics bind to the hydrophobic groove of antiapoptotic BCL‐2 proteins, preventing their interaction with proapoptotic members of the family. Neutralization of antiapoptotic protein activities leads to apoptotic priming [19, 21]. In this sense, ABT‐199 (venetoclax), a selective BCL‐2 inhibitor, has been the first BH3 mimetic approved by FDA for the treatment of chronic lymphoid leukemia (CLL) [22, 23]. ABT‐199 is currently being tested for its possible use in other neoplasms, alone or in combination with other drugs [24, 25, 26]. In this regard, the BELLINI trial reported the benefits of using ABT‐199 in combination with bortezomib and dexamethasone in relapsed/refractory MM patients (NCT02755597) [26]. On the contrary, the search for a safe, effective and selective MCL‐1 inhibitor has been challenging due to the poor pharmacokinetic profile and limited membrane permeability of proposed candidates. Nowadays selective MCL‐1 inhibitors are in various stages of preclinical development, and just a few are being evaluated in clinical trials. In this sense, most relevant MCL‐1 direct inhibitors developed are AZD‐5991, S63845 and its clinical version S64315, AMG 176 and AMG 397 [27]. Concerning S63845, it has been reported that this BH3 mimetic is able to kill MCL‐1‐dependent cancer cells *in vitro*, including leukemia, lymphomas and MM cells [21, 28] and it synergized with ABT‐199 in T‐cell acute lymphoblastic leukemia (T‐ALL) [29]. Furthermore, A‐1155463 is a novel and selective BCL‐X_L_ inhibitor discovered through nuclear magnetic resonance screening [30]. Unlike the observed with ABT‐737, mice thrombocytopenia caused by A‐1155463 seems to be reversible [30]. Accordingly, de Jong et al. [31] reported A‐1155463 synergistic combination with traditional chemotherapy (doxorubicin and cisplatin) in chondrosarcoma and Rello‐Varona et al. [32] confirmed the promising effect of the BCL‐X_L_ inhibitor A‐1331852 combined with dinaciclib in soft‐tissue sarcoma models.

In this article, we have analyzed the mechanism of dinaciclib‐induced apoptosis and evaluated its enhancement by BH3 mimetics (ABT‐199, S63845 or A‐1155463) to improve its anti‐MM activity. We found that combination of dinaciclib with ABT‐199 or A‐1155463 potentiated toxicity mainly in MM cell lines partially dependent on MCL‐1 and also in plasma cells from MM patients, *ex vivo*. Our results open the possibility to further explore CDK9 inhibition as a targeted therapy in MM patients overexpressing or with high dependence on MCL‐1.

## 2 Materials and methods

### 2.1 Reagents and drugs

ABT‐199, A‐1155463, dinaciclib, NVP‐2, palbociclib, S63845 and Z‐VAD‐FMK (hereafter zVAD) were purchased from MedChemExpress (Sollentuna, Sweden). Drug stock solutions were made in DMSO at 1–10 mm concentration. Final DMSO concentration in all experiments was lower than 0.1% according to ISO 10993‐5 for *in vitro* cytotoxicity. Polyethylenimine used in lentiviral generation and polybrene for MM spin infection were both provided by Sigma‐Aldrich (San Luis, MO, USA). Puromycin for Bax and Bak knock‐out (KO) mutant selection was also from Sigma‐Aldrich.

### 2.2 MM cell lines, patient samples and cultures

Human MM cell lines: KMS‐12‐BM (RRID: CVCL_1334), MM.1S (RRID: CVCL_8792), NCI‐H929 (RRID: CVCL_1600), OPM‐2 (RRID: CVCL_1625), RPMI 8226 (RRID: CVCL_0014) and U266 (RIDD: CVCL_0566) all from the ATCC® (Manassas, VA, USA) were routinely cultured in RPMI 1640 + Glutamax medium supplemented with 10% fetal bovine serum (FBS) and 1% penicillin/streptomycin (hereafter, complete medium). RPMI 8226 pLZR, pLZR‐MCL‐1 or pLZR‐BCL‐X_L_ cells were previously generated in our laboratory [33, 34]. HEK‐293T (RRID: CVCL_0063) cell line was cultured in complete DMEM + Glutamax medium or Opti‐MEM™ + l‐Glutamine + HEPES + 2.4 g·L^−1^ NaHCO_3_ medium with 10% FBS (24–48 h for lentiviral generation). All human cell lines have been authenticated by short tandem repeat DNA genotype analysis within the last 3 years and that all experiments were carried out with mycoplasma‐free cells, routinely tested by ‘Mycoplasma Gel Detection Kit’ from Biotools (Madrid, Spain). BM patient samples (*n* = 37) were collected and provided by Hematology Units of Hospital Clínico Universitario ‘Lozano Blesa’ and Hospital Universitario ‘Miguel Servet’. The study was conducted according to the guidelines of the Declaration of Helsinki, and approved by the Ethics Committee of Instituto Aragonés de Ciencias de la Salud (CEICA, protocol code PI16/0129 and date of approval 25 May 2016). Written informed consent was obtained from all subjects involved in the study. Samples were collected from June 2019 to December 2020. BM samples were centrifuged in Ficoll gradient and then BM mononuclear cells (BMMCs) were immediately cultured *ex vivo* for cytotoxic assays in RPMI 1640 + Glutamax medium supplemented with 10% heat‐inactivated FBS and IL‐6 (1000 U·mL^−1^). Patient characteristics and cytogenetics are summarized in Table 1. BMMCs samples were classified into two groups, according to their FISH positivity namely the absence (FISH−) or presence (altered) of routinely tested t(4;14), t(11;14), t(6;14), t(14;16), t(14;20), gain/amplification 1q (+1q), tumor protein P53 (TP53) alterations and *IGH‐FGFR3/IGH‐MAF* gene fusions.

**Table 1:** Clinical characteristic and frequency of cytogenetic determinants of MM patients (*N* = 37). NA, not available. Patients' samples were classified into two groups, according to their FISH positivity namely the absence (FISH−) or presence (altered) of routinely tested t(4;14), t(11;14), t(6;14), t(14;16), t(14;20), gain/amplification 1q (+1q), tumor protein p53 (TP53) alterations and *IGH‐FGFR3/IGH‐MAF* gene fusions.

Characteristic	*n* (%) or *n*/*N* (%)
Age	
Median	75
Range	49–87
PCs, %	
Median	29
Range	1.5–87
Immunoglobulin type	
IgG	19 (51.3)
IgA	2 (5.4)
Bence‐Jones	5 (13.5)
NA	11 (29.8)
Light chain type	
Kappa	14 (37.8)
Lambda	9 (24.4)
NA	14 (37.8)
Cytogenetics	
FISH−	12 (32.4)
Altered	15 (40.6)
NA	10 (27)

### 2.3 Cell viability and cytotoxicity assays

Cell viability of MM cells was routinely evaluated by the Trypan blue exclusion test. MTT assay (performed as described previously [35]) was used to measure cellular metabolic activity as an indicator of cell viability, proliferation and cytotoxicity. For toxicity assays, MM cell lines (3 × 10^5^ cells·mL^−1^) and BMMC samples (10^6^ cells·mL^−1^) were treated in flat bottom, 48‐well plate (500 μL per well) with dinaciclib alone or in combination with BH3 mimetics (ABT‐199, S63845 or A‐1155463) in complete medium for the indicated times (24 h for MM cell lines and 16 h for *ex vivo* samples). Apoptosis was evaluated by determining phosphatidylserine (PS) exposure by flow cytometry.

### 2.4 Western blot analysis

Changes in the amount of cytosolic or mitochondrial proteins were determined by western blot of cell extracts. After treatment with the corresponding drugs (3 × 10^6^ cells per condition), cells were resuspended in 60 μL of lysis buffer [50 mm Tris/HCl pH 7.6 buffer containing 0.15 m NaCl, 10% glycerol, 1 mm Na_3_VO_4_, 1 mm EDTA, 10 mm NaF, 10 μg·mL^−1^ leupeptin, 1 mm phenylmethylsulfonyl fluoride and 1% Triton X‐100] for 30 min on ice. Solubilized proteins were resolved by SDS–15% PAGE and transferred to nitrocellulose membranes. Afterward, membranes were blocked in TBS‐T (10 mm Tris/HCl pH 8.0, 0.12 m NaCl, 0.1 g·L^−1^ thimerosal and 0.1% Tween‐20) containing 5% skimmed milk and incubated with primary antibodies (4 °C, overnight) diluted in TBS‐T containing 5% bovine serum albumin and 0.05% NaN_3_. Primary antibodies anti‐MCL‐1 (sc‐12756 and sc‐819) and anti‐BAK (sc‐832) were provided by Santa Cruz Biotechnology, Inc. (Dallas, TX, USA). Primary anti‐BAX antibody (#2772) and anti‐BCL‐X_L_ (rabbit, polyclonal) were supplied by Cell Signaling Technology (Danvers, MA, USA). Finally, membranes were washed with TBS‐T and incubated with the corresponding peroxidase‐labeled secondary antibodies (Sigma‐Aldrich) in TBS‐T 2.5% skimmed milk and revealed by Amersham™ Imager 600 (GE Healthcare; Chicago, IL, USA). Control of protein loading was achieved by reprobing with anti‐α‐tubulin mAb (clone B‐5‐1‐2; Sigma‐Aldrich). Band quantification was performed using imagej software (Bethesda, MD, USA).

### 2.5 Gene silencing with CRISPR/Cas9

MM.1S cell line was serially spin infected (as described by Bauer et al. [36]) with lentiviral particles (previously generated in HEK‐293T cells transfected with lentiviral vectors: psPAX2, pMD2.G and LentiCRISPR v2 from Addgene; Watertown, MA, USA) for BAX and BAK silencing. LentiCRISPR v2 vector stably expresses Cas9 enzyme, puromycin resistance as well as designed single guided (sgRNA) for BAX and BAK respectively. The target sequences of sgRNAs selected for the gene silencing process were the following: BAK‐sgRNA Fw: 5′‐CACCGGTCCTCCCAGGCAGGAGTG‐3′ and BAK‐sgRNA Rv: 5′‐AAACCACTCCTGCCTGGGAGGAC‐3′; BAX‐sgRNA Fw: 5′‐CACCGGATCGAGCAGGGCGAATGG‐3′ and BAX‐sgRNA Rv: 5′‐AAACCCATTCGCCCTGCTCGATCC‐3′. MM KO cells (or DKO in case of Bax^−/−^ and Bak^−/−^) were selected by culture in media containing puromycin (2–3 μg·mL^−1^) followed by limiting dilution. As a control, MM.1S cells were infected and puromycin selected with lentiviral particles containing empty LentiCRISPR v2 vector (MM.1S Ø). Accordingly, BAX^−/−^, BAK^−/−^ and DKO were confirmed by western blot.

### 2.6 3D cultures of MM cell lines

U266 and MM.1S were cultured in 3D system by using GrowDex™ (UPM Biomedicals, Helsinki, Finland) as a 3D matrix. For that purpose, 6 × 10^4^ cells (U266 and MM.1S) were treated in a flat bottom, 96‐well (200 μL per well) plate with dinaciclib in combination with BH3 mimetics (ABT‐199, S63845 or A‐1155463) in complete medium mixed with GrowDex™ matrix 0.25%. After 24 h of incubation, supernatant was discarded and 20 μL per well of GrowDase™ (also from UPM Biomedicals), mixed with complete RPMI 1640 medium, was added. GrowDase™ was then incubated for 3 h at 37 °C. Finally, samples were filtered through Corning® cell strainers (40 μm pore size), cells stained with annexin V‐FITC/PI and analyzed by flow cytometry.

### 2.7 Flow cytometry

For annexin V staining, cell lines were resuspended in 100 μL of annexin V binding buffer (ABB) containing HEPES/NaOH 10 mm pH 7.4, NaCl 140 mm, CaCl_2_ 2.5 mm and 0.5 μL of annexin V‐FITC per condition. Recombinant annexin V was purified and conjugated with FITC in our laboratory following Logue et al. procedure [37]. In 3D cultures, propidium iodide (PI) (Invitrogen, Carlsbad, CA, USA) was used as necrosis marker. BMMCs obtained from BM patient samples were labeled in 100 μL of ABB with 0.02 μL of annexin V‐DY634 (purified and conjugated with DY634 in our laboratory as mentioned earlier), 1 μL of 7‐AAD as necrosis marker (Biolegend, San Diego, CA, USA) and 4 μL of anti‐CD38‐FITC (clone MI15; BD Pharmigen, San Diego, CA, USA) to specifically identify MM cells. Samples were acquired by BD FACSCalibur or in a BD LSRFortessa™ flow cytometers (Franklin Lakes, NJ, USA) and results were analyzed by BD flowjo 8.6.1 software (Ashland, OR, USA).

### 2.8 Statistical methods

Statistical analysis was performed by using graphpad prism 9.0 software (Dotmatics, Boston, MA, USA). As indicated in each case, data were statistically analyzed by using the following statistical tests: two‐tailed unpaired *t* test for the analysis of two groups for independent samples, two‐tailed paired *t* test for the analysis of two groups with matching or paired samples, one‐way ANOVA with Tukey HSD post‐test for correction of multiple comparisons, when more than two groups were compared. Fisher's exact test was performed to compare contingency tables. The difference between experimentally observed (OBS) specific apoptosis and hypothetical expected (EXP) specific apoptosis, calculated as previously described by other authors [38, 39, 40, 41], was used to determine possible synergies between combinations tested in MM cell lines and plasma cells, *ex vivo*. In particular, it is an approximation of the well‐established method known as Bliss Independence Model in which the combination treatment is considered synergistic if the OBS effect significantly exceeded the EXP effect. EXP specific apoptosis parameter assumes an additive effect of the two combined drugs, and was calculated by using the formula: [(% apoptosis dinaciclib + % apoptosis BH3 mimetic) − (% apoptosis dinaciclib × % apoptosis BH3 mimetic)/100]. In some cases, the Chou‐Talalay method [42] was also utilized (compusyn software, supplied by ComboSyn, Inc., Paramus, NJ, USA) to determine possible synergies. Additionally, receiver operating characteristics (ROC) test was used for validating cut‐off values between patients' samples cohorts.

## 3 Results

### 3.1 Characterization of dinaciclib‐induced apoptosis in MM cell lines

**Fig. 1:**
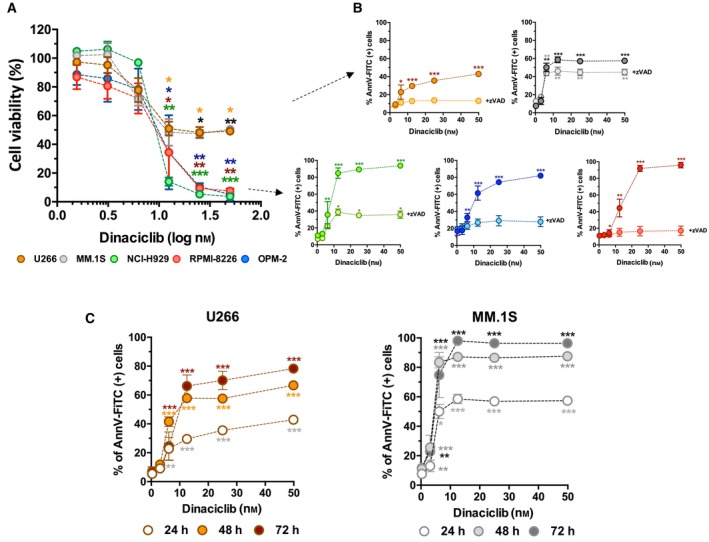
Analysis of dinaciclib‐induced cell death. (A) Analysis of cell proliferation (MTT assay) in MM cell lines incubated with increasing dinaciclib concentrations (24 h). Statistical analysis was performed using two‐tailed unpaired *t* test always comparing to untreated cells (*P < 0.05, **P < 0.01, ***P < 0.001). Data from four independent experiments, global mean and SD are represented. (B) Apoptosis induction in MM cell lines incubated for 24 h with increasing concentrations of dinaciclib in the presence or absence of the pan‐caspase inhibitor Z‐VAD‐FMK (50 μm). Apoptosis was determined by measuring PS exposure through the binding of annexin V‐FITC. Statistical analysis was performed using two‐tailed unpaired *t* test always compared to controls (*P < 0.05, **P < 0.01, ***P < 0.001). Data from four independent experiments, global mean and SD are represented. (C) Time course analysis of dinaciclib toxicity in U266 and MM.1S cell lines. Cells were incubated with increasing concentrations of dinaciclib at different times (24–72 h). Statistical analysis was performed using two‐tailed unpaired *t* test always compared to controls (*P < 0.05, **P < 0.01, ***P < 0.001). Data from four independent experiments, global mean and SD are represented.

Multiple myeloma cells were first incubated with different drug concentrations (0–50 nm) for 24 h. Analysis of cell proliferation showed that U266 and MM.1S cells were more resistant to dinaciclib than NCI‐H929, OPM‐2 and RPMI 8226 (Fig. 1A). These results were confirmed by determining cell apoptosis through annexin V binding analysis (Fig. 1B) and determining lethal dose (LD_50_) (Table S1). Dinaciclib‐induced cell death was notably reduced in all MM cell lines by coincubation with the general caspase inhibitor zVAD (Fig. 1B). After 48 h of incubation dinaciclib became toxic for MM.1S cells, but 72 h incubation were necessary to raise death levels up to 80% in U266 cells (Fig. 1C).

**Fig. 2:**
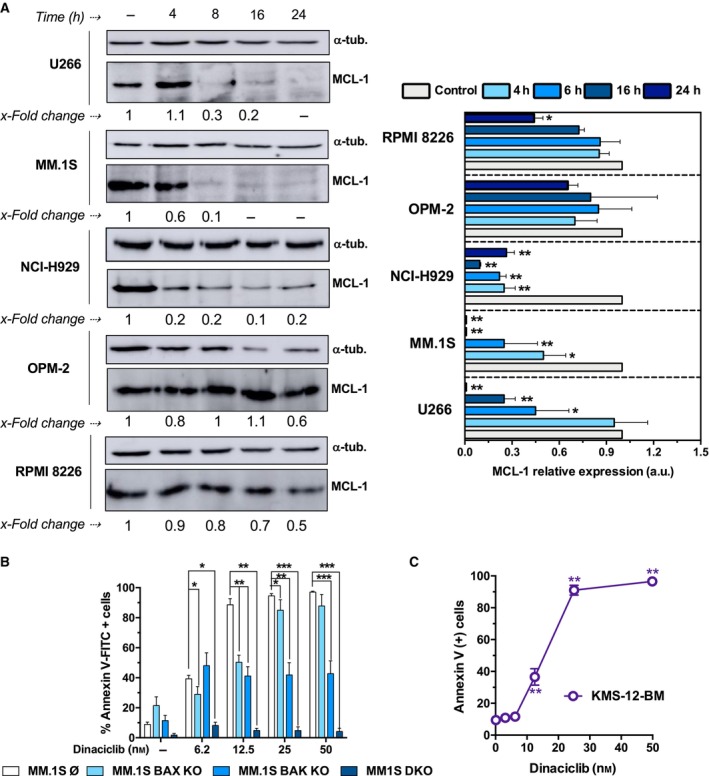
Insights into dinaciclib‐mediated apoptotic mechanism in MM cells. (A) Time course MCL‐1 expression levels in cells incubated with dinaciclib (25 nm for U266 and MM.1S, 15 nm for OPM‐2 and RPMI 8226 and 10 nm for NCI‐H929 cells) during indicated times. Left panel, representative western blot results of dinaciclib‐treated MM cells. Lower numbers indicate MCL‐1 expression (clone sc‐12756) relative to that of α‐tubulin and control situation. Right panel, analysis of western blot results. Statistical analysis was performed using two‐tailed unpaired *t* test always compared to controls (*P < 0.05, **P < 0.01). Data from three independent experiments, global mean and SD are represented. (B) Effect of dinaciclib concentration in genetically modified MM.1S cell lines incubated with the drug for 24 h. Apoptosis was determined by measuring PS exposure through the binding of annexin V‐FITC. Statistical analysis was performed by using one‐way ANOVA test with Tukey's HSD post‐test (*P < 0.05, **P < 0.01, ***P < 0.001). Data from four independent experiments, global mean and SD are represented. (C) Effect of dinaciclib concentration on apoptosis induction in KMS‐12‐BM cells (24 h). Apoptosis was determined by measuring PS exposure through the binding of annexin V‐FITC. Statistical analysis was performed using two‐tailed unpaired *t* test, comparing control to treated situations (**P < 0.01). Data from four independent experiments, global mean and SD are represented.

Mitochondria have a key role in cell death and survival. BAX and BAK are the main proapoptotic proteins of the BCL‐2 family, which turn from harmless monomers into deadly oligomers causing MOMP [43]. As reported previously, MCL‐1 levels decrease as a consequence of CDK9 inhibition [18, 44, 45, 46]. As shown in Fig. 2A, U266 and MM.1S drastically reduced their MCL‐1 expression after 16 h of drug incubation. On the contrary, the decrease in MCL‐1 protein levels was less prominent, but effective, in the case of OPM‐2 and RPMI 8226 cells. NCI‐H929 cells virtually lost MCL‐1 expression after 4 h incubation with dinaciclib and this was associated with the induction of cell death. However, NCI‐H929 survivor cells partially regained basal MCL‐1 levels after 24 h incubation. Single or combined BAX and BAK deletion mutants of MM.1S cells were generated by CRISPR/Cas9 genome editing (Fig. S1A,B) and their sensitivity to dinaciclib determined by annexin V‐FITC binding (Fig. 2B). Results demonstrated that dinaciclib‐induced death mainly occurs through the intrinsic or mitochondrial apoptotic pathway and is more dependent on BAK than on BAX in MM cells (Fig. 2B).

To gain insight into the mechanism of dinaciclib‐induced cell death, we analyzed the drug sensitivity of RPMI 8226 cells overexpressing MCL‐1 or BCL‐X_L_ (Fig. S1C). Results showed a significant protective effect of MCL‐1 and BCL‐X_L_ overexpression in RPMI 8226 cells (Fig. S1D). Dinaciclib‐induced cell death was also evaluated in KMS‐12‐BM cells, a MM cell line previously reported to be a BCL‐2‐dependent MM cell line [47, 48]. KMS‐12‐BM cells were highly sensitive to dinaciclib (Fig. 2C), and similar to the most sensitive cell lines studied.

### 3.2 BH3 mimetics synergize with dinaciclib in MM cell lines

**Fig. 3:**
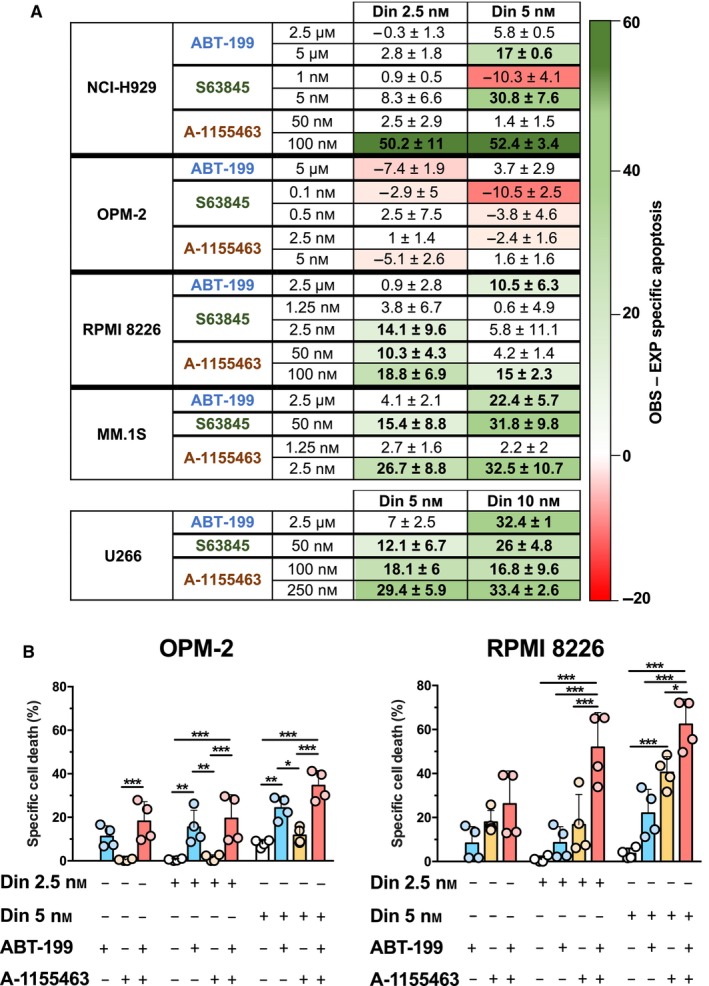
Combinations of dinaciclib and BH3 mimetics in MM cell lines. (A) Cells were incubated with the indicated concentrations of dinaciclib and the corresponding BH3 mimetic (24 h). Single drug concentration for each line was adjusted so that cell death was not greater than 30%. Specific apoptosis was determined by measuring PS exposure through the binding of annexin V‐FITC. The combinations were considered to be synergistic if empirically observed (OBS) − expected (EXP) specific apoptosis was > 10 units. Data from four independent experiments, global mean and SD are indicated. (B) Cell death induced by triple combinations of dinaciclib, ABT‐199 (OPM‐2: 5 μm and RPMI 8226: 2.5 μm) and A‐1155463 (OPM‐2: 5 nm and RPMI 8226: 100 nm). Apoptosis was determined by measuring PS exposure through the binding of annexin V‐FITC. Statistical analysis was performed by using two‐way ANOVA test with Bonferroni post‐test (*P < 0.05, **P < 0.01, ***P < 0.001). Data from four independent experiments, global mean and SD are represented.

We next tried to improve the *in vitro* efficiency of dinaciclib by combining this inhibitor with one BH3 mimetic: ABT‐199 (a BCL‐2 inhibitor), S63845 (a MCL‐1 inhibitor) or A‐1155463 (a BCL‐X_L_ inhibitor). We adjusted single drug concentration for each line so that the amount of cell death was not greater than 30%. The difference between OBS and EXP specific apoptosis for each combination was calculated as indicated in Section 2.8. According to our data, BH3 mimetics ABT‐199, S63845 or A‐1155463 synergized with dinaciclib and potentiated apoptosis in MM cell lines (Fig. 3A, Figs S2 and S3), particularly in U266 and MM.1S cells. These results were confirmed by using the Chou‐Talalay method in U266 and MM.1S cell lines (Fig. S4). In the case of NCI‐H929, OPM‐2 and RPMI 8226 cell lines, a synergistic effect was only observed with some BH3 mimetics, namely when combining dinaciclib with A‐1155463. Interestingly, triple combinations of dinaciclib, ABT‐199 and A‐1155463 (Fig. 3B, Fig. S5) resulted synergistic in the case of RPMI 8226 cells, indicating that direct targeting of BCL‐2 and BCL‐X_L_ and indirect inhibition of MCL‐1 could overcome double combination resistance in this cell line.

We also analyzed the effect of dinaciclib combinations with BH3 mimetics in 3D culture models generated with the cell lines more resistant to dinaciclib (U266 and MM.1S). Dinaciclib combinations with BH3 mimetics were quite efficient in promoting cell death in these 3D models, as illustrated in Fig. S6A,B. The combinations of dinaciclib with ABT‐199 or A‐1155463 led to greater toxicity in both lines tested.

### 3.3 CDK9 inhibition mediates synergy of dinaciclib‐based combinations with BH3 mimetics

**Fig. 4:**
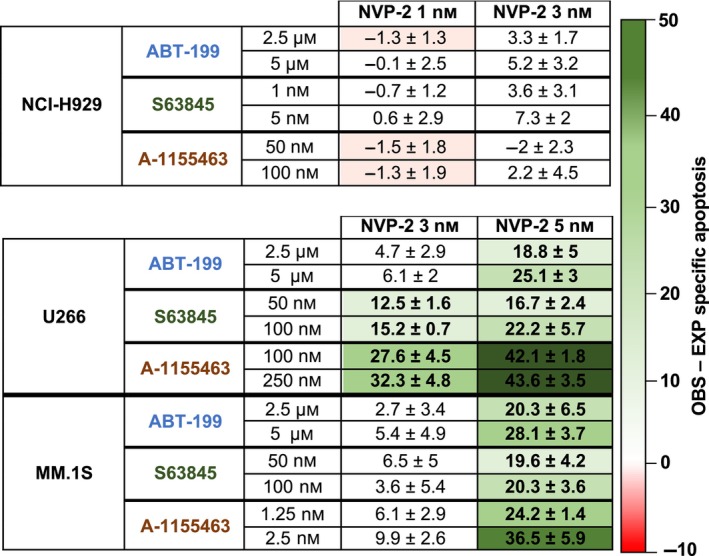
Combinations of dinaciclib and NVP‐2 (CDK9 inhibitor) in MM cell lines. Cells were incubated with the indicated concentrations of dinaciclib and NVP‐2 (24 h). Single drug concentration for each line was adjusted so that cell death was not greater than 30%. Specific apoptosis was determined by measuring PS exposure through the binding of annexin V‐FITC. The combinations were considered to be synergistic if empirically observed (OBS) − expected (EXP) specific apoptosis was > 10 units. Data from three independent experiments, global mean and SD are indicated.

CDK9 activity regulates mRNA transcription [46, 49, 50]. When CDK9 activity is inhibited by dinaciclib, protein turnover of short‐life cell proteins such as MCL‐1 is impaired. In this regard, the specific CDK9 inhibitor NVP‐2 [51] was also used. Combinations of NVP‐2 and BH3 mimetics efficiently synergized in cell lines partially dependent on MCL‐1 (U266 and MM.1S) as already observed with dinaciclib (Fig. 4, Fig. S7). To better evaluate the role of CDK9 in dinaciclib‐based combinations with BH3 mimetics, the effect of an alternative CDK inhibitor, palbociclib, which inhibits CDK4 and 6 but not CDK9 [52], was analyzed. Results indicated that apoptotic potency of palbociclib was much lower than that of dinaciclib after 24 h incubation (Fig. S8A). Cell death in U266 cells was only achieved after 48 h incubation with 10 μm palbociclib and 72 h incubation were needed to induce death in MM.1S and NCI‐H929 cells. Furthermore, palbociclib‐treated cells seemed to acquire a senescent‐like phenotype with bigger size and internal microvesiculation (Fig. S8B).

### 3.4 Sensitivity of primary MM cells to dinaciclib action

**Fig. 5:**
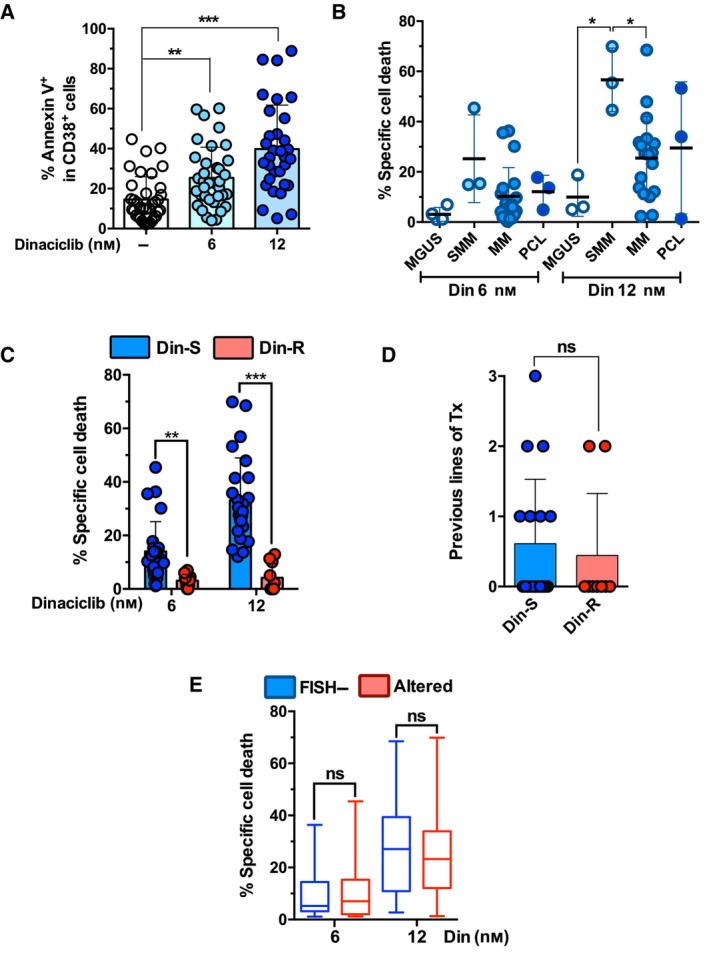
Effect of incubation of the indicated dinaciclib concentrations with plasma cells from MM patients for 16 h, *ex vivo*. (A) Dinaciclib‐induced apoptosis in patient samples. Apoptosis was determined by measuring PS exposure through the binding of annexin V‐DY634 in CD38 positive cells and analyzed by flow cytometry. Statistical analysis was performed using two‐tailed unpaired *t* test (**P < 0.01, ***P < 0.001). Global mean and SD are illustrated (*n* = 37). (B) Dinaciclib‐induced specific cell death of samples attending to their disease stage [MGUS (*n* = 3–4); SMM (*n* = 3); MM (*n* = 23–27); PCL (*n* = 3)]. Specific apoptosis was determined by measuring PS exposure through the binding of annexin V‐DY634 in CD38 positive cells and analyzed by flow cytometry. Statistical analysis was performed using one‐way ANOVA with Tukey's HSD post‐test (*P < 0.05). Global mean and SD in each cohort are illustrated. (C) Individual *ex vivo* sensitivity of plasma cells from MM patients to increasing dinaciclib concentrations, classifying samples according to their resistance (Din‐R, *n* = 10) or sensitivity (Din‐S, *n* = 27) to dinaciclib. Cut‐off values were validated by ROC analysis. Statistical analysis was performed using two‐tailed unpaired *t* test (**P < 0.01, ***P < 0.001). Global mean and SD in each cohort are illustrated. (D) Number of previous lines of treatment of MM patients were analyzed according to their sensitivity to dinaciclib (Din‐S, *n* = 18; Din‐R, *n* = 9). Statistical analysis was performed using two‐tailed unpaired *t*‐test (ns, nonsignificant). Global mean and SD in each cohort are illustrated. (E) Specific dinaciclib‐induced cell death in Din‐S and Din‐R cohorts according to their cytogenetic status: FISH− (*n* = 12) or altered (*n* = 15). Statistical analysis was performed using two‐tailed unpaired *t* test (ns, nonsignificant). Global mean and SD in each cohort are illustrated.

The *ex vivo* sensitivity to dinaciclib of plasma cells from MM patients was analyzed (*n* = 37). As illustrated in Fig. 5A, plasma cells from MM patients were as sensitive to dinaciclib action as MM cell lines. Apoptotic rates were dose dependent, reaching a mean value of almost 40% of annexin V^+^ cells with 12 nm dinaciclib. This concentration was close to the LD_50_ of the majority of the MM cell lines tested (Table S1). We observed a great variability in dinaciclib sensitivity among patients as has been recurrently observed with other types of drugs in MM [53, 54]. We also determined the sensitivity to dinaciclib depending on disease stage of patient samples (Fig. 5B). Plasma cells from MGUS, MM and PCL patients displayed lower sensitivity to low (6 nm) dinaciclib concentration than samples from SMM patients; however, the differences among them were not statistically significant. At a higher concentration (12 nm), samples from MGUS patients were significantly less sensitive than those of SMM, MM or PCL (Fig. 5B).

Patient samples were also subdivided into two groups, dinaciclib‐sensitive (Din‐S) and dinaciclib‐resistant (Din‐R) according to their *ex vivo* drug response. We considered as Din‐R those samples showing a death rate in the lower quartile of death values displayed by all tested samples (specific apoptosis lower than 5.8% and 14.2% for 6 and 12 nm respectively) and Din‐S those whose death rates were upper than the first quartile. Both cut‐off values were validated by ROC analysis (Table S2). As illustrated in Fig. 5C, Din‐S cohort exhibited a significant response to treatment with dinaciclib alone, and, as expected, Din‐R patients showed a lower response to dinaciclib, *ex vivo*.

We next analyzed the possible correlation between clinical parameters of MM patients and the *ex vivo* sensitivity to dinaciclib. No significant differences were found between Din‐S and Din‐R subgroups regarding the previous lines of treatment (Fig. 5D). We also analyzed the *ex vivo* response of plasma cells to dinaciclib depending on the existence of cytogenetic alterations. BMMC samples were classified into two groups, according to their FISH positivity, namely the absence or presence of t(4;14), t(11;14), t(6;14), t(14;16), t(14;20), gain/amplification 1q (+1q), TP53 alterations and *IGH‐FGFR3/IGH‐MAF* gene fusions. In this regard, no significant differences were found between both groups, suggesting that sensitivity to dinaciclib is independent of the presence of these recurrent MM cytogenetic alterations (Fig. 5E).

### 3.5 Sensitivity of plasma cells to BH3 mimetics and role of MCL‐1 and BCL‐2 in dinaciclib‐induced cell death

**Fig. 6:**
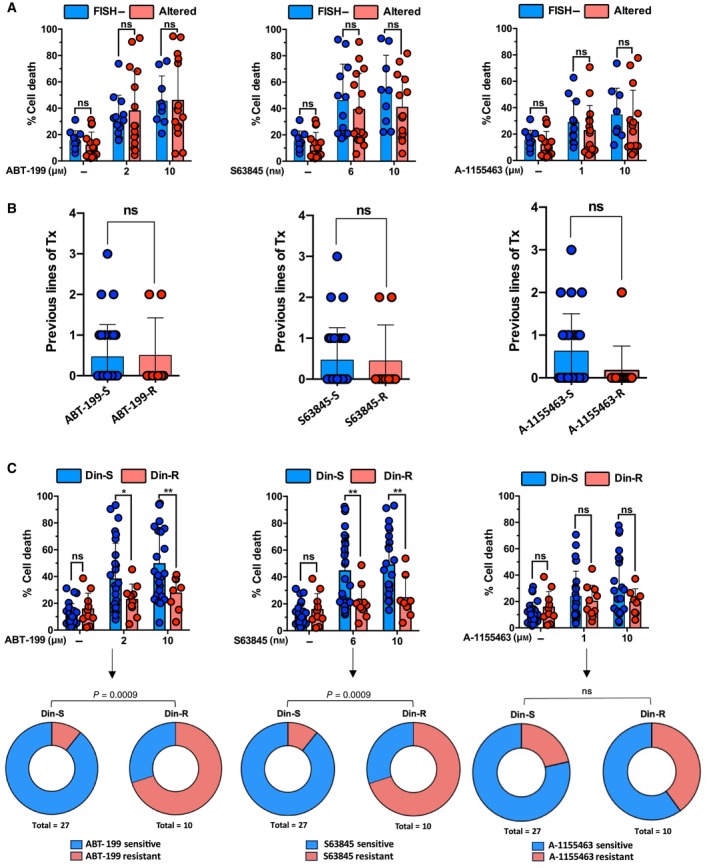
Effect of BH3 mimetics in plasma cells from MM patients. (A) BH3 mimetics were incubated at the indicated concentrations for 16 h with plasma cells from MM patients and cell death was determined by flow cytometry. Sensitivity to BH3 mimetics was evaluated according to their cytogenetic status: FISH− (*n* = 12) or altered (*n* = 15). Statistical analysis was performed using one‐way ANOVA with Tukey's HSD post‐test (ns, nonsignificant). Global mean value and SD in each cohort are illustrated. (B) Previous lines of treatment that MM patients were compared according to their sensitivity to single BH3 mimetic‐induced death. Sensitive (S): those samples whose BH3 mimetic‐induced cell death was higher than the first quartile (ABT‐199, *n* = 28; S63845, *n* = 28 and A‐1155463, *n* = 24) and resistant (R): those samples whose BH3 mimetic‐induced cell death was in the lower quartile (ABT‐199, *n* = 8; S63845, *n* = 9 and A‐1155463, *n* = 12). Statistical analysis was performed using two‐tailed unpaired *t* test (ns, nonsignificant). Global mean and SD in each cohort are illustrated. (C) Upper panel: *Ex vivo* sensitivity to BH3 mimetics of plasma cells from patients according to their sensitivity to dinaciclib (Din‐S, *n* = 10; Din‐R, *n* = 27). Statistical analysis was performed using two‐tailed unpaired *t* test (*P < 0.05, **P < 0.01; ns, nonsignificant). Global mean and SD in each cohort are illustrated. Lower panel: Toxicity of the corresponding BH3 mimetic in plasma cells (sensitive or resistant), classified according to their response to dinaciclib (Din‐S or Din‐R). Statistical analysis was performed using Fisher's exact *t* test (ns, nonsignificant).

We first studied the effect of single BH3 mimetics in plasma cells from MM patients, *ex vivo*. No significant differences were found when comparing the response to ABT‐199, S63845 or A‐1155463 in plasma cells with FISH‐negative or altered cytogenetics (Fig. 6A). Nevertheless, FISH− samples tend to be more sensitive to S63845 than altered ones, but this tendency did not reach statistical significance due to the wide range of responses. The number of previous lines of treatment did not significantly influence *ex vivo* plasma cells response to BH3 mimetics (Fig. 6B), though resistance to A‐1155463 was mainly found in patients that have received a higher number of previous lines of treatment.

Results obtained with MM cell lines suggested that sensitivity to dinaciclib was related to dependence on MCL‐1. In order to extend the validity of this observation to plasma cells from patients, we analyzed the sensitivity to BH3 mimetics of Din‐R and Din‐S cohorts. As illustrated in Fig. 6C, Din‐R samples were less sensitive to S63845, suggesting again the critical role of MCL‐1 in dinaciclib‐induced cell death. Moreover, Din‐R samples were also more resistant to ABT‐199, which could indicate that dependence on BCL‐2 could also determine the *ex vivo* sensitivity to dinaciclib.

### 3.6 Sensitivity of plasma cells to dinaciclib combinations with BH3 mimetics

**Fig. 7:**
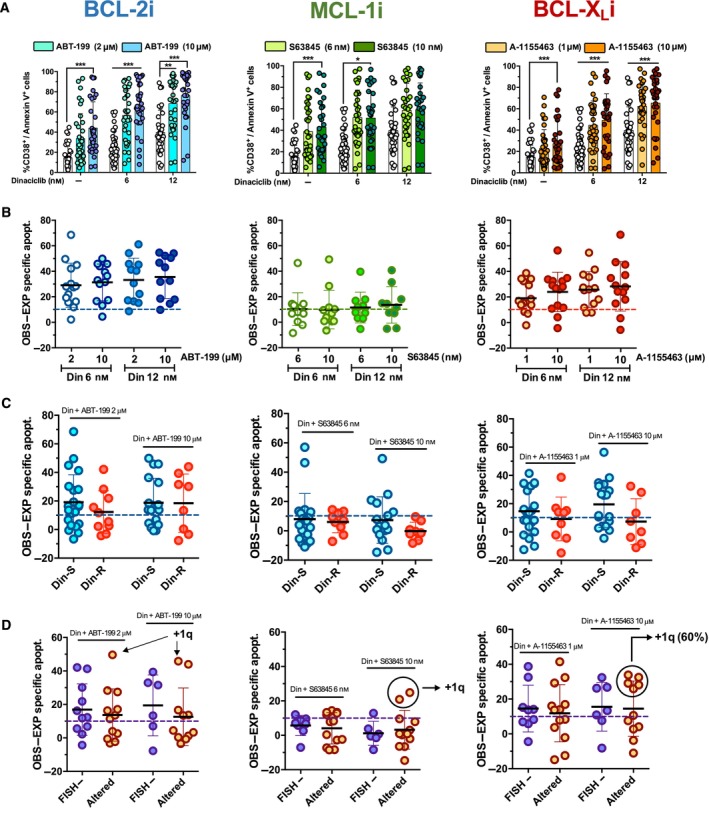
*Ex vivo* toxicity of combinations of dinaciclib with BH3 mimetics on plasma cells from MM patients. Plasma cells from MM samples were incubated with two different doses of dinaciclib combined with two different doses of each BH3 mimetic for 16 h and apoptosis evaluated through PS exposure determined by annexin V‐DY634 binding in CD38 positive population. (A) Global response of plasma cells to dinaciclib and BH3 mimetics combinations. Statistical analysis was performed using one‐way ANOVA with Tukey's HSD post‐test (*P < 0.05, **P < 0.01, ***P < 0.001). Global mean and SD of 37 independent samples are illustrated. (B) Empirically observed (OBS) − expected (EXP) in those MM samples whose cell death was prompted as a consequence of dinaciclib and BH3 mimetics combination (ABT‐199, *n* = 14; S63845, *n* = 12; A‐1155463, *n* = 16). The combinations were considered to be synergistic if OBS − EXP specific apoptosis was > 10 units (represented with dashed line). Global mean and SD are illustrated. (C) Analysis of toxicity of dinaciclib‐based combinations based on the resistance or sensitivity to single‐dinaciclib action (16 h). OBS − EXP specific apoptosis of dinaciclib (6 nm)‐based combinations with ABT‐199, S63845 or A‐1155463 are shown. Specific apoptosis was determined by measuring PS exposure through the binding of annexin V‐DY634 in CD38 positive cells. The combinations were considered to be synergistic if OBS − EXP specific apoptosis was > 10 units (represented with dashed line). Global mean and SD in each cohort are illustrated (Din‐S, *n* = 27; Din‐R, *n* = 10). (D) Analysis of toxicity of dinaciclib‐based drug combinations according to patients' cytogenetic status: FISH− (*n* = 6–12) or altered (*n* = 11–12) for 16 h. OBS − EXP values of dinaciclib (6 nm)‐based combinations with ABT‐199, S63845 or A‐1155463 are shown. Specific apoptosis was determined by measuring PS exposure through the binding of annexin V‐DY634 in CD38 positive cells. The combinations were considered to be synergistic if OBS − EXP specific apoptosis was > 10 units (represented with dashed line). Global mean and SD in each cohort are illustrated.

Since combination of dinaciclib and BH3 mimetics showed synergism in MM cell lines, we next analyzed whether this was also the case in plasma cells from myeloma patients. As illustrated in Fig. 7A, concurrent treatment with dinaciclib and BH3 mimetics, at two different drug concentrations, further increased CD38^+^/annexin V^+^ population compared to single treatment with dinaciclib, especially when in combinations with ABT‐199 or A‐1155463. Synergy was determined by calculating EXP specific apoptosis and subsequent difference between OBS − EXP, as done with established cell lines. Accordingly, plasma cells were classified into three different groups: first, those samples that were sensitive to dinaciclib or single BH3 mimetic treatment (EXP specific apoptosis was in the upper quartile of each combination analysis); second, samples showing moderate apoptotic rates with each single drug that were potentiated by drug combinations, and, finally, insensitive cells to combinations of dinaciclib with BH3 mimetics (OBS and EXP cell death values were similar and in the lower quartile). The difference between OBS and EXP specific apoptosis was just calculated for samples classified in the second group. As shown in Fig. 7B, the cytotoxicity of dinaciclib in plasma cells, *ex vivo*, significantly increased when combined with ABT‐199 or A‐1155463. We could observe clear *ex vivo* synergy in 13/36 (36.1%) samples for ABT‐199 or A‐1155463 dinaciclib‐based combinations, and in 6/35 (17.1%) samples for S63845 combinations.

We would also like to analyze whether the resistance or sensitivity to dinaciclib treatment *ex vivo* could correlate with the response to combinations. As in the previous analysis, we only considered samples that were not sensitive to single drugs but displayed a positive synergy to combinations between dinaciclib and BH3 mimetics. As illustrated in Fig. 7C and Fig. S9, combination of dinaciclib and ABT‐199 led to the most significant results. In case of dinaciclib and A‐1155463, an additive effect was observed in Din‐R group, and a synergistic effect was observed in Din‐S samples. Finally, and according to our previous results, the combined effect of dinaciclib and S63845 was mainly additive or antagonistic in both cohorts.

As previously mentioned, MM samples were classified into two groups attending to their cytogenetic status. Again, for this analysis, we only considered samples that did not show high sensitivity to single drugs. In this sense, no statistically significant differences between patients with FISH negative or altered cytogenetics were found in the response to combinations of dinaciclib with BH3 mimetics (Fig. 7D). Interestingly, best response values in the altered group were normally reached by samples bearing 1q21 amplification (+1q), a high‐risk cytogenetic alteration recurrently observed in MM patients [55]. Just one patient bearing +1q was classified as Din‐R (Table S3).

## 4 Discussion

Throughout the last decades, a great improvement of the myeloma therapeutic toolkit has occurred. The introduction of targeted drugs, such as proteasome inhibitors and cell therapies resulted in an increase of the median overall survival [56]. However, MM relapse and acquired drug resistance will result in limited long‐term survival in many cases [57]. Consequently, novel targeted drugs or improved drug combinations are needed. In this report, we have delved into the mechanism of apoptosis induced by dinaciclib, a CDK inhibitor with robust antimyeloma activity [4] as well as its possible improvement by combination with BH3 mimetics, a direct‐acting apoptosis inducers [58]. We found that dinaciclib induced caspase‐dependent apoptosis in different MM cell lines and especially in those dependent on MCL‐1 (NCI‐H929, OPM‐2 and RPMI 8226). U266 and MM.1S cells, depending only partially on MCL‐1, as shown by S63845 titration [59, 60], were more resistant to dinaciclib toxicity. In this case, downregulation of MCL‐1 protein (caused by CDK9 inhibition) does not critically impair the cellular balance between pro‐ and antiapoptotic proteins. Furthermore, overexpression of MCL‐1 in RPMI 8226 cells, which are supposed to be dependent on MCL‐1 [60], drastically reduced dinaciclib‐induced cell death. Moreover, by using a BAX and BAK DKO MM cell line we have demonstrated that dinaciclib‐induced death occurs through the intrinsic apoptotic pathway, according to previous studies in mouse embryonic fibroblasts and melanoma cells [61, 62]. Our results also confirmed that dinaciclib‐induced apoptosis seems to depend more on BAK than on BAX, as previously reported for melanoma cells [62]. For this reason, it would be interesting to evaluate combinations of dinaciclib with drugs which are reported to be dependent on BAX [e.g. inhibitors of heat shock protein 90 (HSP90) or histone deacetylases (HDACs) inhibitors] [63] to boost synergistic effect.

Interestingly, we observed that dinaciclib synergizes with BH3 mimetics binding to BCL‐2 or BCL‐X_L_ and increases apoptosis rates in some MM cell lines and plasma cells from MM patients, *ex vivo*. This has been previously found with other CDK inhibitors and BH3 mimetics. In particular, Zhou et al. [64] found synergic effect of flavopiridol combined with a BCL‐2 inhibitor in MM cells and Rello‐Varona et al. [32] found a high apoptosis induction by combination of dinaciclib with an inhibitor of BCL‐X_L_ in soft‐tissue sarcomas. The combination of dinaciclib with ABT‐199 has also been proposed against acute lymphocytic leukemia [65].

It is well‐known that CDK9 plays a main role in the control of basal gene transcription, thus assuring cell transcriptional homeostasis [49]. Previous studies have shown that CDK9 inhibition negatively regulates MYC as well as MCL‐1 and BCL‐2 expression [50, 66]. In this sense, a study with 60 MM patient samples demonstrated that 63% of them responded to the *ex vivo* inhibition of MCL‐1 [59] and MCL‐1 overexpression and dependence on this protein is a bad prognostic factor for MM patients [67]. When constitutive transcription is blocked by a CDK9 inhibitor (such as dinaciclib), short‐lived proteins, such as MCL‐1, decrease quickly [18, 27, 44]. Dinaciclib combinations with ABT‐199 or A‐1155463 were effective in MM cell lines that were partially dependent on MCL‐1, probably due to the simultaneous inactivation of MCL‐1 (by dinaciclib) and other antiapoptotic proteins (by BH3 mimetics). This was confirmed by using the specific CDK9 inhibitor NVP‐2 and correlates with previous studies combining dinaciclib with iz‐huTRAIL in which simultaneous downregulation of MCL‐1 and cFLIP_L_ was involved in observed synergy [68]. The main difference between dinaciclib and NVP‐2 is that while dinaciclib inhibits CDK1, 2, 5 and 9, NVP‐2 is a specific CDK9 inhibitor. Since we hypothesized that CDK9 was essential for observed synergy, the specificity of NVP‐2 for CDK9 should enhance the synergy observed in U266 and MM.1S cell lines. Nevertheless, the observed synergy in NCI‐H929 cells was notably reduced compared with that of dinaciclib, which would suggest that CDK1, 2 or 5 inhibition will also favoring the synergy between dinaciclib and BH3 mimetics.

Moreover, Din‐R group of plasma cells from MM patients exhibited low sensitivity to S63845, according to the proposed link between dependence on MCL‐1 and dinaciclib sensitivity. Additionally, partial dependence on BCL‐2 could also be related to the sensitivity to dinaciclib, as we found in our samples from patients. Within this context, we also observed that KMS‐12‐BM cell line, considered to be highly dependent on BCL‐2 [47], was sensitive to dinaciclib. Since CDK9 is also involved in BCL‐2 expression [50], blocking CDK9 activity could also affect to BCL‐2‐dependent cells.

Multiple myeloma genome instability not only facilitates clonal proliferation and spread of disease, but also creates cell vulnerabilities which can be used to develop new therapeutic strategies. In this study, we have evaluated if cytogenetic alterations were associated to dinaciclib response in MM cells. In this sense, we could not observe any significant correlation between genetic aberrations and response in MM cells, either in cell lines or in plasma cells from patients. However, plasma cells displaying +1q were highly sensitive to dinaciclib, alone or in combination with BH3 mimetics. 1q21 amplification is one of the most common cytogenetic alterations occurring in around 40% of *de novo* patients and 70% of relapsed/refractory patients [55]. The presence of this alteration is correlated with high sensitivity to MCL‐1 inhibitors since chromosome 1q is where MCL‐1 locus resides [69], and it agrees to our hypothesis that dependence on MCL‐1 correlates with dinaciclib sensitivity. Eventually, the presence of +1q alteration could be used as a biomarker to carry out dinaciclib‐based clinical trials. Interestingly, we observed that MGUS samples seemed to be less sensitive to dinaciclib 12 nm than those of SMM, MM or PCL. Our work is the first to evaluate the sensitivity to MCL‐1 indirect inhibitor depending on disease stage of MM and so it is not possible to compare with previous works. In particular, it has been reported that in MGUS the prevalence of +1q is lower than in SMM or MM patients, as determined by FISH and next‐generation sequencing techniques [70, 71, 72, 73]. This observation could explain differences in sensitivity to dinaciclib (an indirect MCL‐1 inhibitor) among samples, and the low sensitivity to dinaciclib observed in MGUS samples.

## 5 Conclusions

The future of MM treatment relies on precision medicine, with decision making based on the *ex vivo* analysis of particular characteristics of malignant plasma cells. In this sense, dynamic BH3 profiling (DBP) has emerged as a useful tool to identify cellular dependence on individual antiapoptotic proteins with a simple FACS analysis or a microfluidic‐based DBP device [74, 75]. As an alternative, standardized titration of cancer cells sensitivity to BH3 mimetics may be addressed. In particular, BH3 mimetics standardized titration could be useful to determine the dependence of plasma cells to MCL‐1. In case of dependence on MCL‐1, CDK9 inhibition could be a suitable therapy through reduction of MCL‐1 protein levels and cell viability [46]. This observation opens the possibility to explore CDK9 inhibition as a targeted therapy in MM patients overexpressing or with high dependence on MCL‐1 and therefore poor prognosis [67]. Combinations of CDK9 inhibitors with ABT‐199 or A‐1155463 may be used in case of MM cells that co‐depend on BCL‐2 or BCL‐X_L_ respectively. In this regard, reversible thrombocytopenia caused by A‐1155463 [30] may be prevented by using improved BCL‐X_L_ inhibitors [76]. To achieve this, the search for more specific CDK9 inhibitors would be also crucial to reduce related side effects observed in the clinic. They could be a surrogate to MCL‐1 inhibitors, which may cause cardiac toxicity [77, 78, 79]. It will be interesting to clinically determine possible adverse effects occasioned by the combination of these drugs. In this sense, there are still no clinical results about this type of combinations among CDKs inhibitors and BH3 mimetics. However, there are some ongoing clinical trials combining fadraciclib (multi‐CDK2, 9 inhibitor) and ABT‐199 against relapsed/refractory CLL (NCT03739554), relapsed/refractory acute myeloid leukemia and myelodysplastic syndrome patients (NCT04017546) [80] which will shed light on this field. All in all, combinations between CDK9 inhibitors and other myeloma targeted drugs (proteasome inhibitors) or cellular therapies [chimeric antigen receptor T cells] could be worthy explored.

## Conflict of interest

The authors declare no conflict of interest.

## Author contributions

MB‐V, JN and IM designed the study; MB‐V, NJ‐A, MC, AS‐DV and AB performed sample analysis; MB‐V, VP, AA, JN and IM interpreted and analyzed data; RD, GA and LP collected samples, reviewed patient records and collected data; MB‐V and IM wrote the manuscript. All authors had full access to all data, carefully reviewed the manuscript and approved the final version.

### Peer review

The peer review history for this article is available at https://www.webofscience.com/api/gateway/wos/peer‐review/10.1002/1878‐0261.13522.

## Supporting information


**Fig. S1.** Validation and functional characterization of engineered MM cell lines.

**Fig. S2.** Analysis of cell death induced by combinations of dinaciclib and BH3 mimetics in MM cell lines.

**Fig. S3.** OBS and EXP specific apoptosis induced by dinaciclib‐based combinations with BH3 mimetics in MM cell lines.

**Fig. S4.** Synergy validation between dinaciclib and BH3 mimetics in U266 and MM.1S cell lines by CI calculation through the Chou‐Talalay formula.

**Fig. S5.** OBS–EXP specific apoptosis for triple combinations of dinaciclib, ABT‐199 and A‐1155463 in OPM‐2 and RPMI 8226 cells.

**Fig. S6.** Cell death induced by dinaciclib and BH3 mimetics combinations in MM cell lines partially dependent on MCL‐1 in 3D culture models.

**Fig. S7.** Cell death induced by combinations of NVP‐2 (CDK9 inhibitor) and BH3 mimetics in MM cell lines.

**Fig. S8.** Effect of CDK4/6 inhibition on MM cell lines.

**Fig. S9.** Cell death induced by dinaciclib‐based combinations with BH3 mimetics in Din‐S and Din‐R subgroups of patients' samples.

**Table S1.** *In vitro* dinaciclib lethal dose (LD_50_) in established multiple myeloma (MM) cell lines used in this study.

**Table S2.** Validation of dinaciclib‐resistant (Din‐R) and dinaciclib‐sensitive (Din‐S) cut‐off values: ROC analysis.

**Table S3.** Summary of samples with high‐risk cytogenetic alterations used in this work. Dinaciclib resistance (Din‐R) or sensitivity (Din‐S) of each sample is also indicated.

## Data Availability

The data supporting in manuscript are available in the article and [Link], [Link], [Link], [Link], [Link], [Link], [Link], [Link], [Link], [Link], [Link], [Link]. All other data are available from the corresponding author upon reasonable request.
