# Supplementary material for: Dinaciclib synergizes with BH3 mimetics targeting BCL‐2 and BCL‐XL in multiple myeloma cell lines partially dependent on MCL‐1 and in plasma cells from patients
Source: Mol Oncol. 2023 Sep 28;17(12):2507–25. doi: 10.1002/1878-0261.13522 (PMC10701777; doi:10.1002/1878-0261.13522)
Supplement: Supplementary file 1 — Fig. S1. Validation and functional characterization of engineered MM cell lines. [file MOL2-17-2507-s006.pdf]

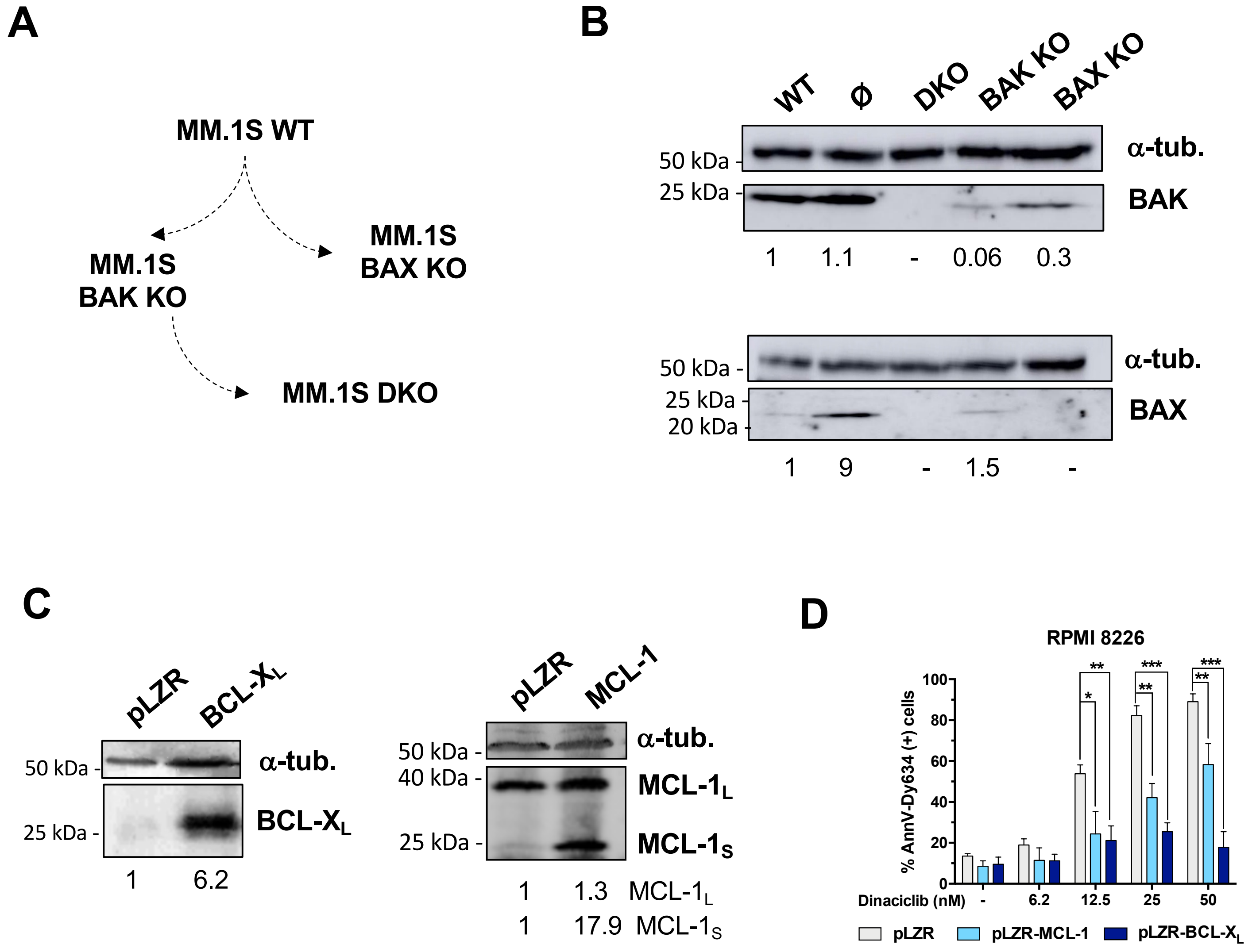

Figure S1. Engineered multiple myeloma (MM) cell lines. (**A**) Short description of the MM.1S strategy to genetically modify MM.1S BAX$^{-/-}$, BAK$^{-/-}$ and BAX$^{-/-}$ and BAK$^{-/-}$ (DKO) cells. MM.1S cell line was serially spin-infected with lentiviral particles. Afterwards, MM.1S KO cells (or DKO in case of BAX$^{-/-}$ and BAK$^{-/-}$) were selected by culture in media containing puromycin (2-3 µg/ml) followed by limiting dilution. As a control, MM.1S cells were infected and puromycin-selected with lentiviral particles containing empty LentiCRISPR v2 vector (MM.1S Ø). (**B**) Accordingly, BAX and BAK expression was analyzed by western blot. Representative images are shown. BAX and BAK levels were quantified by densitometry analysis of bands using ImageJ software and normalized to those of α-tubulin and control (n=2). (**C**) Expression of BCL-X$_L$ (polyclonal antibody) and MCL-1 (clone sc-819) in RPMI 8226 pLZR, RPMI 8226-BCL-X$_L$ and RPMI 8226-MCL-1 cells was analyzed by western blot. Representative images are shown. Bands were quantified by densitometry analysis of bands using ImageJ software and normalized to those of α-tubulin and control (n=2). (**D**) Effect of increasing dinaciclib concentrations in RPMI 8226 cells overexpressing MCL-1 or BCL-XL incubated for 24 h. Apoptosis was determined by measuring phosphatidyl serine (PS) exposure through the binding of annexin V-DY634. Statistical analysis was performed using one-way ANOVA test with Tukey HSD post-test (*p<0.05, **p<0.01, ***p<0.001). Global mean and SD of 3 independent experiments are illustrated.
