# Supplementary material for: Dinaciclib synergizes with BH3 mimetics targeting BCL‐2 and BCL‐XL in multiple myeloma cell lines partially dependent on MCL‐1 and in plasma cells from patients
Source: Mol Oncol. 2023 Sep 28;17(12):2507–25. doi: 10.1002/1878-0261.13522 (PMC10701777; doi:10.1002/1878-0261.13522)
Supplement: Supplementary file 2 — Fig. S2. Analysis of cell death induced by combinations of dinaciclib and BH3 mimetics in MM cell lines. [file MOL2-17-2507-s008.pdf]

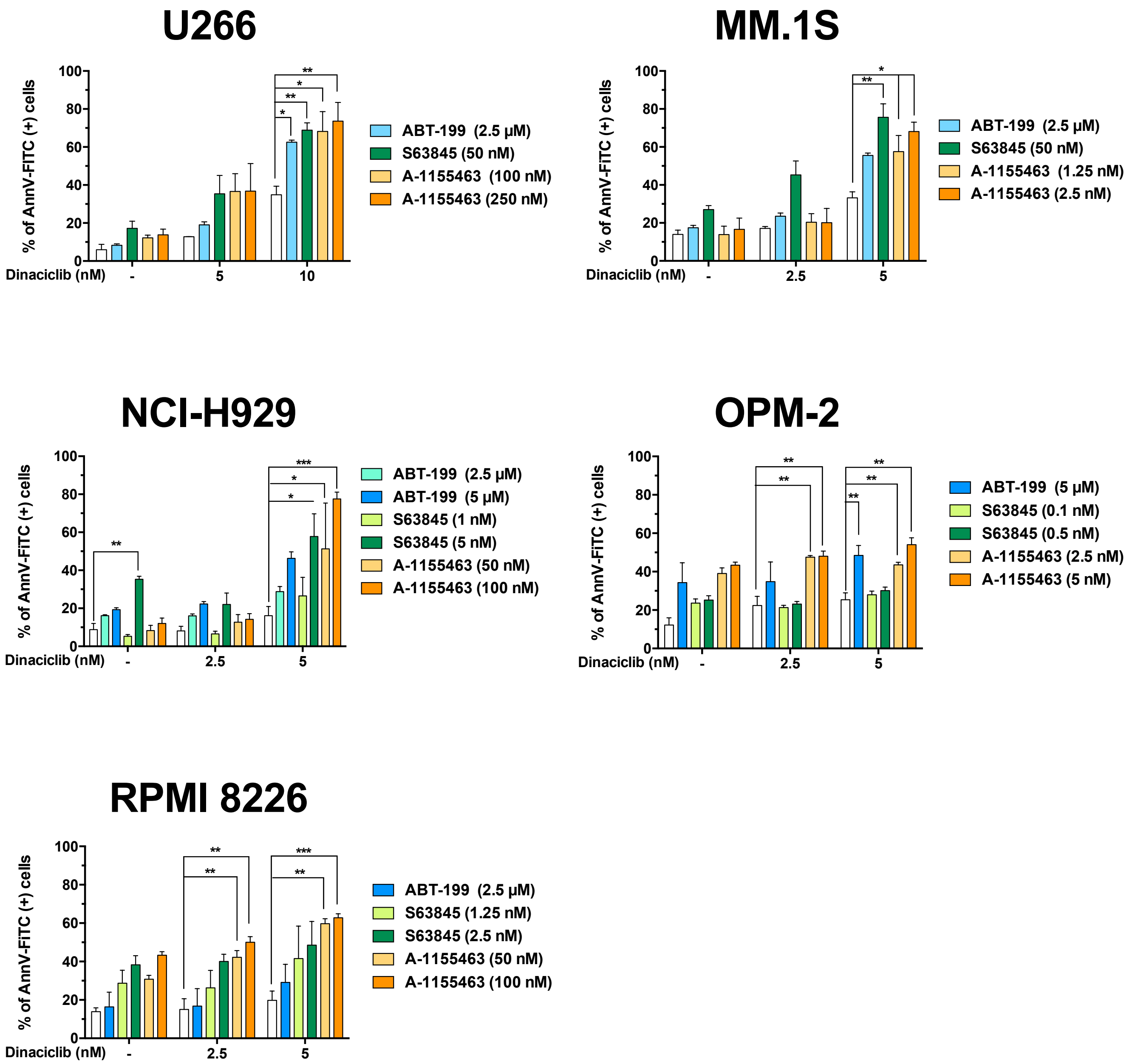

Figure S2. Cell death induced by combinations of dinaciclib and BH3 mimetics in MM cell lines. Cells were incubated with the indicated concentrations of dinaciclib and the corresponding BH3 mimetic for 24 h and cell death was analysed by flow cytometry. Statistical analysis was performed using two-tailed unpaired t-test comparing treated cells to controls (*p<0.05, **p<0.01, ***p<0.001). Global mean and SD of 3-4 independent experiments are illustrated.
