# Supplementary material for: Dinaciclib synergizes with BH3 mimetics targeting BCL‐2 and BCL‐XL in multiple myeloma cell lines partially dependent on MCL‐1 and in plasma cells from patients
Source: Mol Oncol. 2023 Sep 28;17(12):2507–25. doi: 10.1002/1878-0261.13522 (PMC10701777; doi:10.1002/1878-0261.13522)
Supplement: Supplementary file 3 — Fig. S3. OBS and EXP specific apoptosis induced by dinaciclib‐based combinations with BH3 mimetics in MM cell lines. [file MOL2-17-2507-s001.pdf]

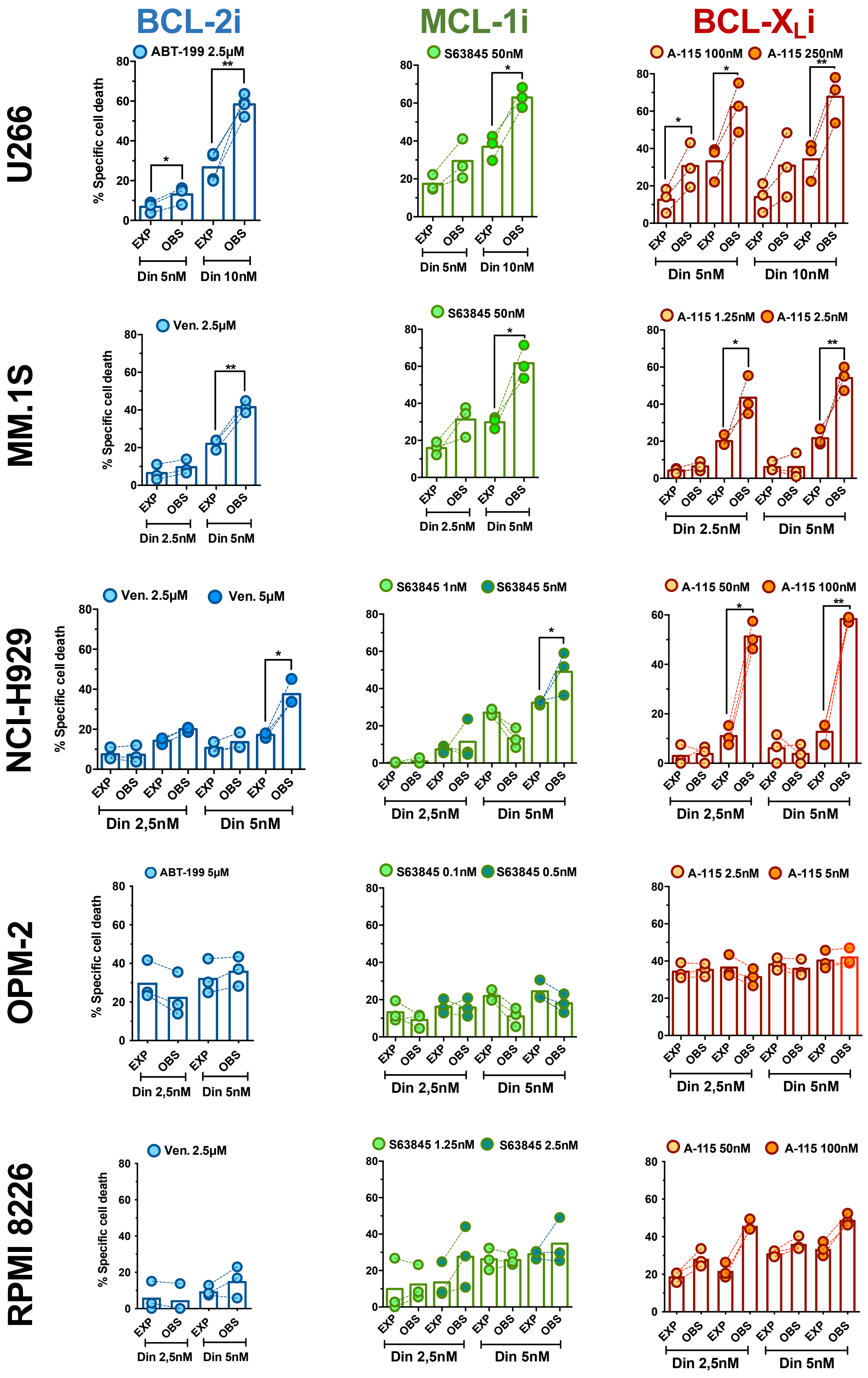

BCL-2i
MCL-1i
BCL-XLi
U266
MM.1S
NCI-H929
OPM-2
RPMI 8226
% Specific cell death
ABT-199 2.5µM
S63845 50nM
A-115 100nM
A-115 250nM
A-115 1.25nM
A-115 2.5nM
Ven. 2.5µM
Ven. 5µM
S63845 1nM
S63845 5nM
A-115 50nM
ABT-199 5µM
S63845 0.1nM
S63845 0.5nM
A-115 5nM
S63845 1.25nM
S63845 2.5nM
EXP
OBS
Din 5nM
Din 10nM
Din 2.5nM
Din 2,5nM

Figure S3. Cells death induced by dinaciclib-based combinations with BH3 mimetics in MM cell lines. Cells were incubated with the indicated concentrations of dinaciclib and the corresponding BH3 mimetic for 24 h and cell death was analysed by flow cytometry. EXP and OBS specific apoptosis of each dinaciclib-based combination with BH3 mimetics are represented. Statistical analysis was performed by using two-tailed paired t-test (*$p<0.05$, **$p<0.01$). Global mean of 3-4 independent experiments is illustrated.
