# Supplementary material for: Dinaciclib synergizes with BH3 mimetics targeting BCL‐2 and BCL‐XL in multiple myeloma cell lines partially dependent on MCL‐1 and in plasma cells from patients
Source: Mol Oncol. 2023 Sep 28;17(12):2507–25. doi: 10.1002/1878-0261.13522 (PMC10701777; doi:10.1002/1878-0261.13522)
Supplement: Supplementary file 4 — Fig. S4. Synergy validation between dinaciclib and BH3 mimetics in U266 and MM.1S cell lines by CI calculation through the Chou‐Talalay formula. [file MOL2-17-2507-s007.pdf]

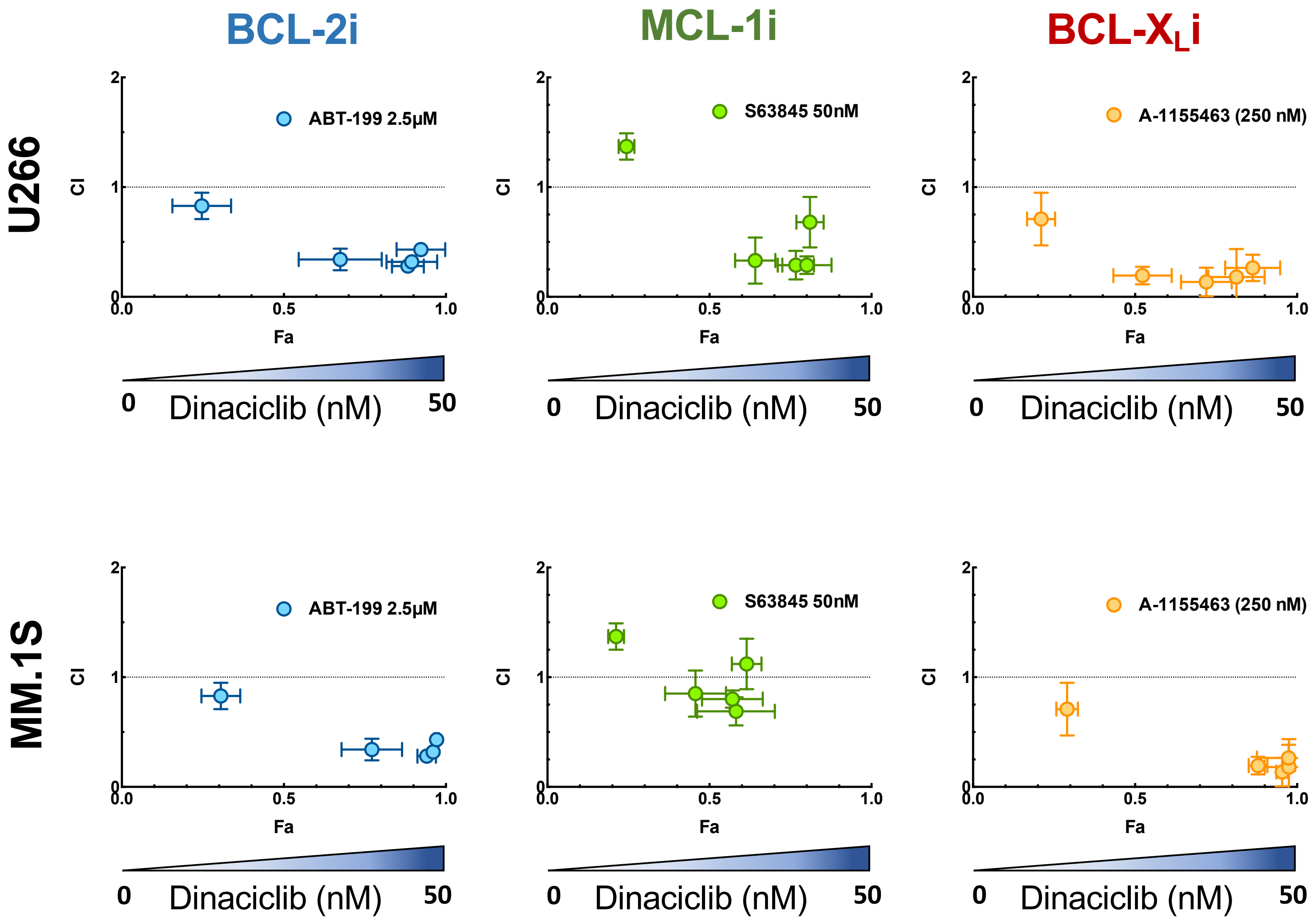

Figure S4. Synergy validation. The Chou-Talalay formula was performed to determine synergy between dinaciclib and BH3 mimetics in myeloid cell leukemia sequence 1 (MCL-1) partially-dependent cell lines. For this purpose, a dose-response curve of dinaciclib with or without the addition of a constant concentration of a BH3 mimetic was performed. The results represent the combination index (CI) value for each combination and the % of death cells (Fa) in each case. Data from 3 independent experiments, global mean and SD are illustrated.
