# Supplementary material for: Dinaciclib synergizes with BH3 mimetics targeting BCL‐2 and BCL‐XL in multiple myeloma cell lines partially dependent on MCL‐1 and in plasma cells from patients
Source: Mol Oncol. 2023 Sep 28;17(12):2507–25. doi: 10.1002/1878-0261.13522 (PMC10701777; doi:10.1002/1878-0261.13522)
Supplement: Supplementary file 5 — Fig. S5. OBS–EXP specific apoptosis for triple combinations of dinaciclib, ABT‐199 and A‐1155463 in OPM‐2 and RPMI 8226 cells. [file MOL2-17-2507-s002.pdf]

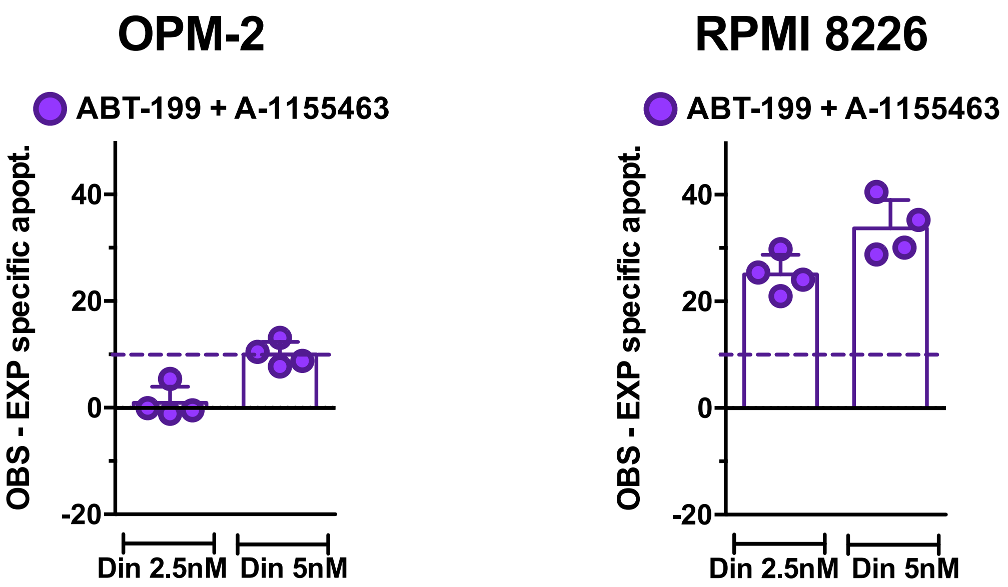

Figure S5. Empirically observed (OBS) – expected (EXP) specific apoptosis for triple combinations. Triple combinations of dinaciclib, ABT-199 (OPM-2: 5 µM and RPMI 8226: 2.5 µM) and A-1155463 (OPM-2: 5 nM and RPMI 8226: 100 nM) were performed as described in Figure 3. The combination ABT-199 + A-1155463 was considered as a single agent, to properly calculate EXP cell death. The combinations were synergistic if OBS-EXP specific apoptosis was greater than 10 units (represented with dashed line). Data from 4 independent experiments, global mean and SD are illustrated.
