# Supplementary material for: Dinaciclib synergizes with BH3 mimetics targeting BCL‐2 and BCL‐XL in multiple myeloma cell lines partially dependent on MCL‐1 and in plasma cells from patients
Source: Mol Oncol. 2023 Sep 28;17(12):2507–25. doi: 10.1002/1878-0261.13522 (PMC10701777; doi:10.1002/1878-0261.13522)
Supplement: Supplementary file 6 — Fig. S6. Cell death induced by dinaciclib and BH3 mimetics combinations in MM cell lines partially dependent on MCL‐1 in 3D culture models. [file MOL2-17-2507-s005.pdf]

A

			Din 20 nM	Din 25 nM
U266	ABT-199	2.5 µM	37.9 ± 1.2	27.5 ± 16.8
		5 µM	37.3 ± 9.4	27.9 ± 17.2
	S63845	50 nM	30.8 ± 9.2	25.5 ± 9
		100 nM	31.5 ± 9.6	18.2 ± 7.1
	A-1155463	100 nM	44 ± 8.2	41.6 ± 8.4
		250 nM	42.1 ± 12.3	37.9 ± 11.8

			Din 8 nM	Din 10 nM
MM.1S	ABT-199	2.5 µM	13.8 ± 10.3	23.5 ± 7.3
		5 µM	14.1 ± 8.1	23.5 ± 11.2
	S63845	50 nM	15 ± 10.8	22.7 ± 6.8
		100 nM	12.1 ± 5.6	21.6 ± 11.1
	A-1155463	1.25 nM	6.8 ± 1.4	22.4 ± 2.2
		2.5 nM	15.9 ± 3.8	28 ± 9.6

OBS – EXP specific apoptosis: 50 / 25 / 0

B

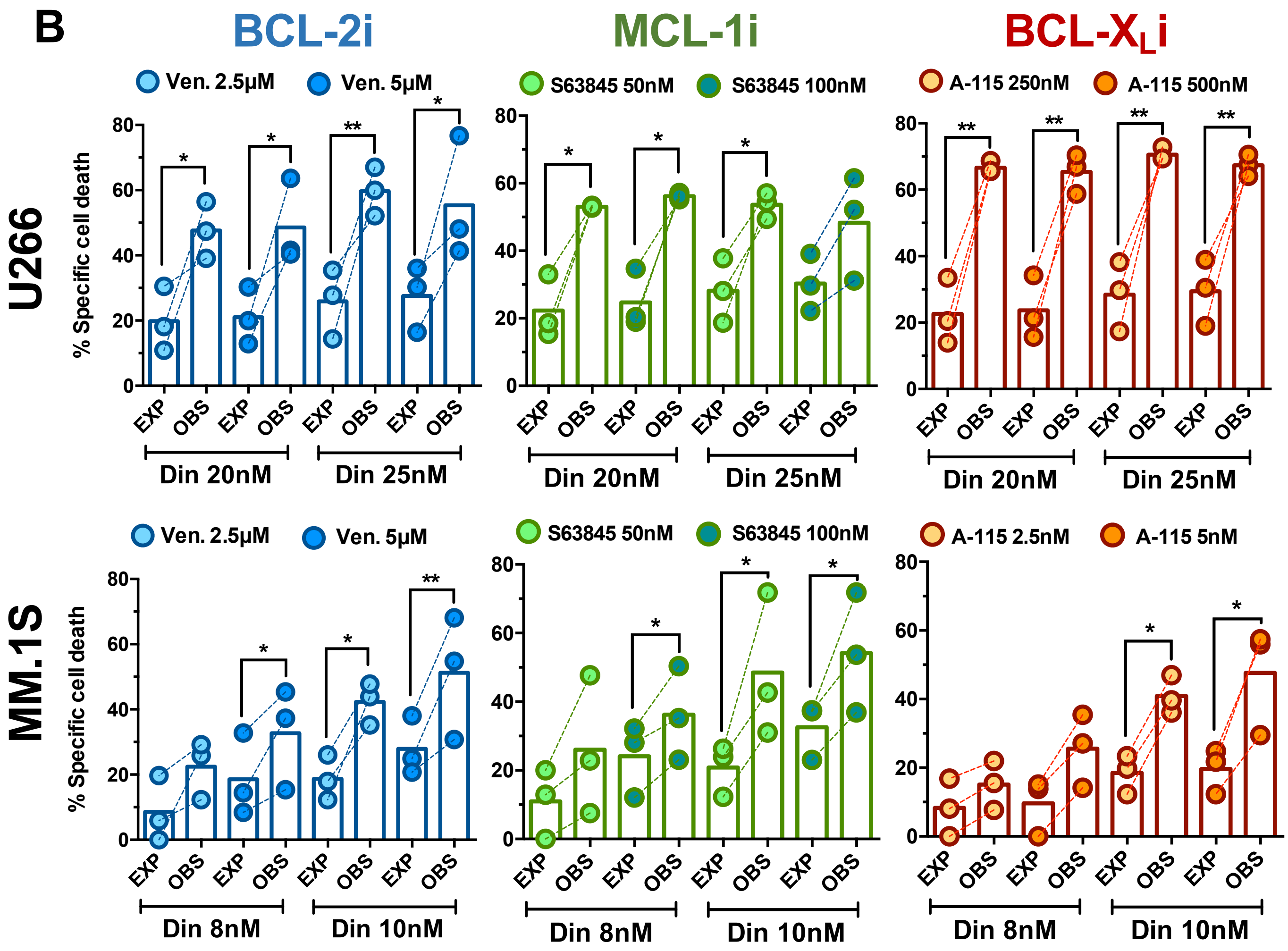

Figure S6. Cell death induced by dinaciclib and BH3 mimetics combinations in multiple myeloma (MM) cell lines partially-dependent on myeloid cell leukemia sequence 1 (MCL-1) in 3D culture models. (**A**) Combinations of dinaciclib and BH3 mimetics in 3D scaffold GrowDex™. Cells were incubated with the indicated concentrations of dinaciclib and the corresponding BH3 mimetic for 24 h. Single drug concentration for each line was adjusted so that cell death was not greater than 30%. Specific apoptosis was determined by measuring phosphatidyl serine (PS) exposure through the binding of annexin V-FITC and propidium iodide (PI) as necrosis marker. The combinations were synergistic if empirically observed (OBS) – expected (EXP) specific apoptosis was greater than 10 units. OBS-EXP specific apoptosis global mean and SD of 3 independent experiments are indicated. (**B**) EXP and OBS specific apoptosis values of each dinaciclib-based combination with BH3 mimetics in 3D culture models are represented. Statistical analysis was performed by using two-tailed paired t-test (*$p<0.05$, **$p<0.01$). Data from 3 independent experiments and global mean are illustrated.
