# Supplementary material for: Dinaciclib synergizes with BH3 mimetics targeting BCL‐2 and BCL‐XL in multiple myeloma cell lines partially dependent on MCL‐1 and in plasma cells from patients
Source: Mol Oncol. 2023 Sep 28;17(12):2507–25. doi: 10.1002/1878-0261.13522 (PMC10701777; doi:10.1002/1878-0261.13522)
Supplement: Supplementary file 7 — Fig. S7. Cell death induced by combinations of NVP‐2 (CDK9 inhibitor) and BH3 mimetics in MM cell lines. [file MOL2-17-2507-s009.pdf]

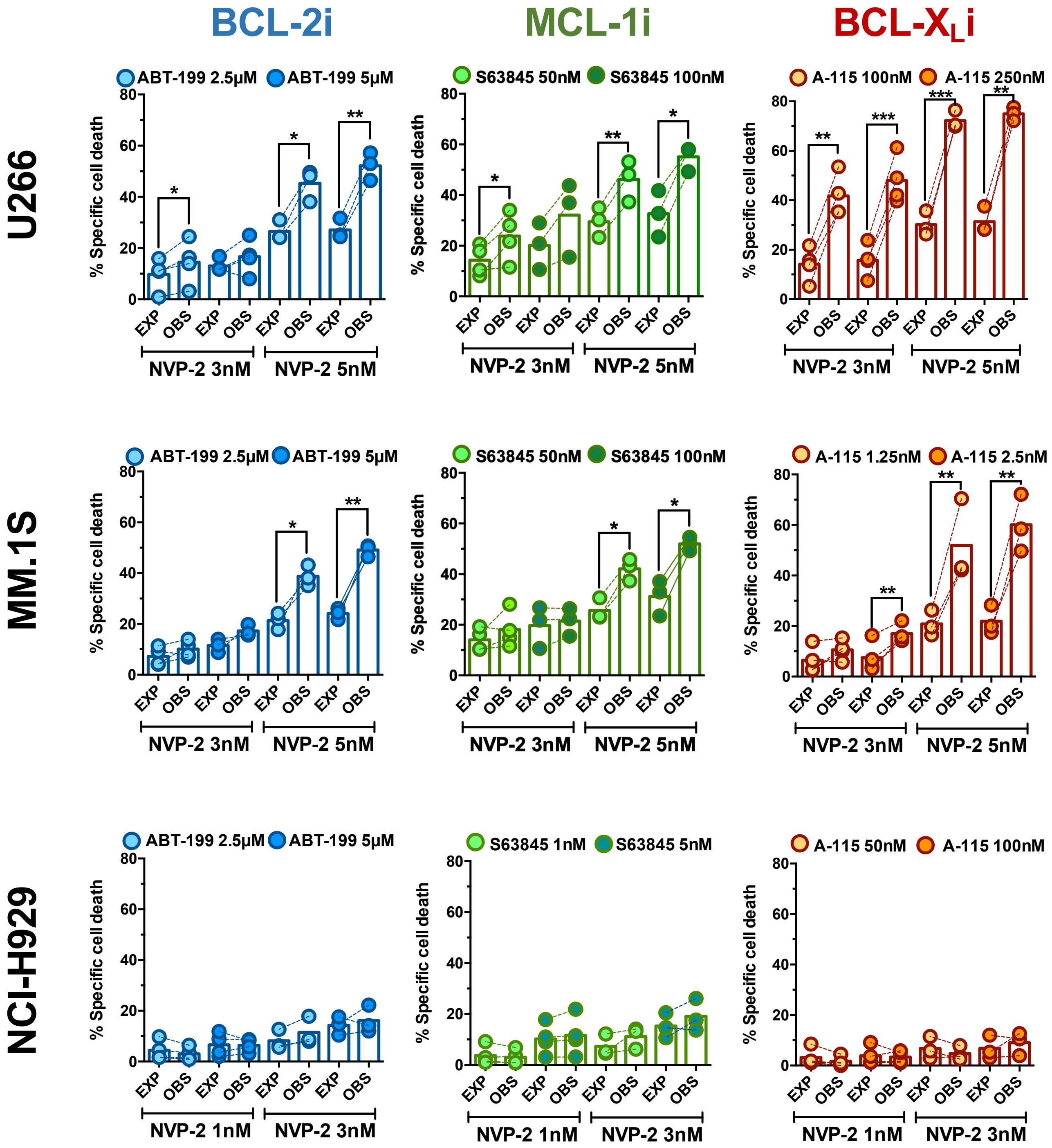

Figure S7. Cell death induced by combinations of NVP-2 (CDK9 inhibitor) and BH3 mimetics in multiple myeloma (MM) cell lines for 24 h. Empirically observed (OBS) and expected (EXP) specific apoptosis values of NVP-2-based combinations with BH3 mimetics are represented. Statistical analysis was performed by using two-tailed paired t-test (*p<0.05, **p<0.01, ***p<0.001). Data from 3-4 independent experiments and global mean are illustrated.
