# Supplementary material for: Dinaciclib synergizes with BH3 mimetics targeting BCL‐2 and BCL‐XL in multiple myeloma cell lines partially dependent on MCL‐1 and in plasma cells from patients
Source: Mol Oncol. 2023 Sep 28;17(12):2507–25. doi: 10.1002/1878-0261.13522 (PMC10701777; doi:10.1002/1878-0261.13522)
Supplement: Supplementary file 8 — Fig. S8. Effect of CDK4/6 inhibition on MM cell lines. [file MOL2-17-2507-s004.pdf]

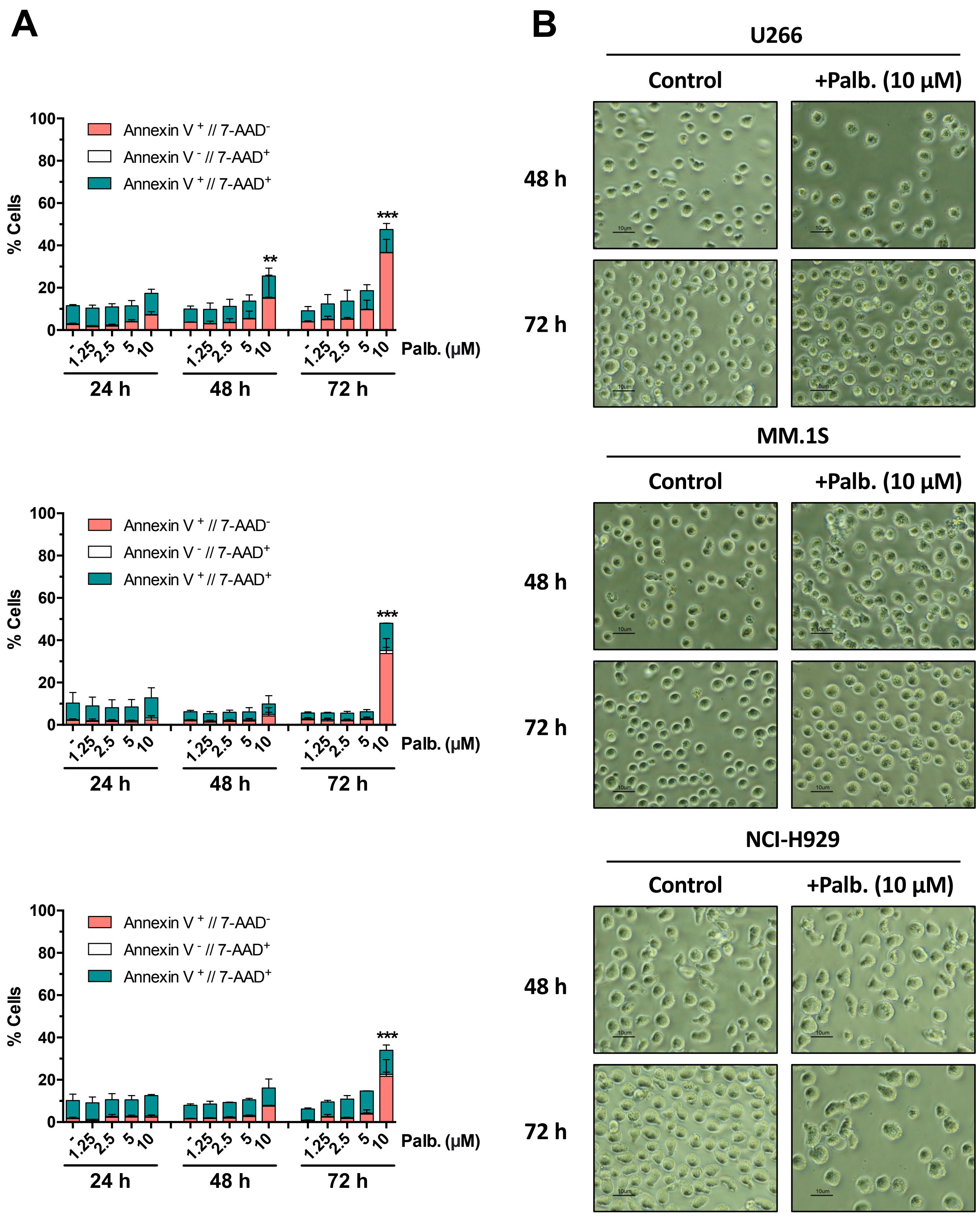

Figure S8. Effect of CDK4/6 inhibition on MM cell lines. (**A**) Palbociclib titration analysis (24, 48 and 72 h) in U266, MM.1S and NCI-H929 cell lines. Statistical analysis was performed by using one-way ANOVA test with Tukey HSD post-test (**p<0.01, ***p<0.001). Data from 3 independent experiments, global mean and SD are illustrated. **(B)** Representative optical inverted microscopy (400X) images of U266, MM.1S and NCI-H929 cells incubated (or not) with palbociclib during 48 or 72 h.
