# Supplementary material for: Dinaciclib synergizes with BH3 mimetics targeting BCL‐2 and BCL‐XL in multiple myeloma cell lines partially dependent on MCL‐1 and in plasma cells from patients
Source: Mol Oncol. 2023 Sep 28;17(12):2507–25. doi: 10.1002/1878-0261.13522 (PMC10701777; doi:10.1002/1878-0261.13522)
Supplement: Supplementary file 9 — Fig. S9. Cell death induced by dinaciclib‐based combinations with BH3 mimetics in Din‐S and Din‐R subgroups of patients' samples. [file MOL2-17-2507-s012.pdf]

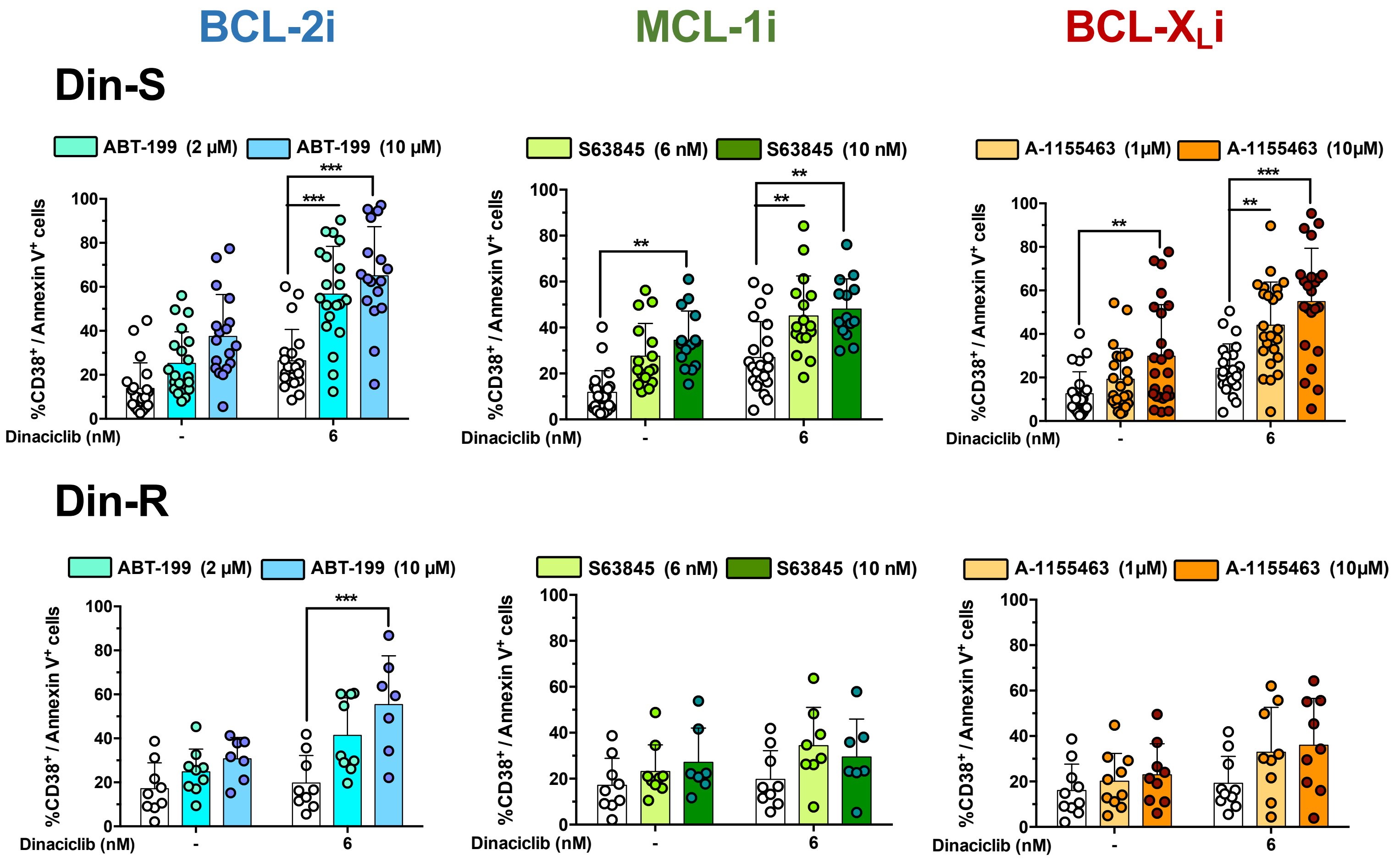

Figure S9. Cell death induced by dinaciclib-based combinations with BH3 mimetics in dinaciclib sensitive (Din-S) and dinaciclib resistant (Din-R) subgroups (24 h). Data from samples that were not sensitive to single drugs but displayed a positive synergy to combinations between dinaciclib and BH3-mimetics are represented (n=9-16). Statistical analysis was performed using one-way ANOVA with Tukey HSD post-test (**p<0.01, ***p<0.001). Global mean and SD are illustrated.
