# Supplementary material for: Dinaciclib synergizes with BH3 mimetics targeting BCL‐2 and BCL‐XL in multiple myeloma cell lines partially dependent on MCL‐1 and in plasma cells from patients
Source: Mol Oncol. 2023 Sep 28;17(12):2507–25. doi: 10.1002/1878-0261.13522 (PMC10701777; doi:10.1002/1878-0261.13522)
Supplement: Supplementary file 10 — Table S1. In vitro dinaciclib lethal dose (LD50) in established multiple myeloma (MM) cell lines used in this study. [file MOL2-17-2507-s003.docx]

**Supplementary Table 1**

**Table S1.** *In vitro* dinaciclib lethal dose **(**LD_50_) in established multiple myeloma (MM) cell lines used in this study.

| **Cell Line** | **LD_50_ (nM) ± SD** |
| --- | --- |
| **KMS-12-BM** | 14.7 ± 3.4 |
| **MM.1S** | 19.6 ± 6.1 |
| **NCI-H929** | 8.9 ± 2.1 |
| **OPM-2** | 9.2 ± 3.7 |
| **RPMI 8226** | 8.2 ± 2.2 |
| **U266** | 25 ± 4.4 |
