# Supplementary material for: Dinaciclib synergizes with BH3 mimetics targeting BCL‐2 and BCL‐XL in multiple myeloma cell lines partially dependent on MCL‐1 and in plasma cells from patients
Source: Mol Oncol. 2023 Sep 28;17(12):2507–25. doi: 10.1002/1878-0261.13522 (PMC10701777; doi:10.1002/1878-0261.13522)
Supplement: Supplementary file 11 — Table S2. Validation of dinaciclib‐resistant (Din‐R) and dinaciclib‐sensitive (Din‐S) cut‐off values: ROC analysis. [file MOL2-17-2507-s011.docx]

**Supplementary Table 2**

**Table S2.** Validation of dinaciclib resistant (Din-R) and dinaciclib sensitive (Din-S) cut-off values: ROC analysis.

| **Dinaciclib 6 nM** | | **Dinaciclib 12 nM** | |
| --- | --- | --- | --- |
| **Area under the ROC curve** | **Results** | **Area under the ROC curve** | **Results** |
| **Area** | 0.847 | **Area** | 0.994 |
| **Std. Error** | 0.064 | **Std. Error** | 0.009 |
| **95% Confidence interval** | 0.722 – 0.97 | **95% Confidence interval** | 0.976 – 1.0 |
| **P value** | 0.0014 | **P value** | <0.0001 |
