# Supplementary material for: Dinaciclib synergizes with BH3 mimetics targeting BCL‐2 and BCL‐XL in multiple myeloma cell lines partially dependent on MCL‐1 and in plasma cells from patients
Source: Mol Oncol. 2023 Sep 28;17(12):2507–25. doi: 10.1002/1878-0261.13522 (PMC10701777; doi:10.1002/1878-0261.13522)
Supplement: Supplementary file 12 — Table S3. Summary of samples with high‐risk cytogenetic alterations used in this work. Dinaciclib resistance (Din‐R) or sensitivity (Din‐S) of each sample is also indicated. [file MOL2-17-2507-s010.docx]

**Supplementary Table 3**

**Table S3**. Summary of samples with high-risk cytogenetic alterations used in this work. Dinaciclib resistance (Din-R) or sensitivity (Din-S) of each sample is also indicated.

| **Sample Code** | **Cytogenetic**  **alteration** | **Dinaciclib response** |
| --- | --- | --- |
| **#3** | +1q | Din-S |
| **#6** | +1q | Din-R |
| **#13** | +1q | Din-S |
| **#16** | +1q | Din-S |
| **#17** | +1q | Din-S |
| **#21** | Mutant TP53 | Din-S |
| **#23** | +1q & t(14;16) | Din-S |
| **#27** | +1q | Din-S |
